# Context-dependent genomic locus effects on antibody production in recombinant Chinese hamster ovary cells generated through random integration

**DOI:** 10.1016/j.csbj.2024.04.023

**Published:** 2024-04-10

**Authors:** Hyun Jee Woo, Jaehoon Kim, Seul Mi Kim, Dongwoo Kim, Jae Yun Moon, Daechan Park, Jae Seong Lee

**Affiliations:** a Department of Molecular Science and Technology, Ajou University, Suwon 16499, Republic of Korea; b Molecular Science and Technology Research Center, Ajou University, Suwon 16499, Republic of Korea; c Department of Biological Sciences, Ajou University, Suwon 16499, Republic of Korea; d Department of Applied Chemistry and Biological Engineering, Ajou University, Suwon 16499, Republic of Korea

**Keywords:** Chinese hamster ovary (CHO), Cell line development, Hot spot, Random integration

## Abstract

High-yield production of therapeutic protein using Chinese hamster ovary (CHO) cells requires stable cell line development (CLD). CLD typically uses random integration of transgenes; however, this results in clonal variation and subsequent laborious clone screening. Therefore, site-specific integration of a protein expression cassette into a desired chromosomal locus showing high transcriptional activity and stability, referred to as a hot spot, is emerging. Although positional effects are important for therapeutic protein expression, the sequence-specific mechanisms by which hotspots work are not well understood. In this study, we performed whole-genome sequencing (WGS) to locate randomly inserted vectors in the genome of recombinant CHO cells expressing high levels of monoclonal antibodies (mAbs) and experimentally validated these locations and vector compositions. The integration site was characterized by active histone marks and potential enhancer activities, and clustered regularly interspaced short palindromic repeats (CRISPR)/CRISPR-associated protein 9 (Cas9) mediated indel mutations in the region upstream of the integration site led to a significant reduction in specific antibody productivity by up to 30%. Notably, the integration site and its core region did not function equivalently outside the native genomic context, showing a minimal effect on the increase in exogenous protein expression in the host cell line. We also observed a superior production capacity of the mAb expressing cell line compared to that of the host cell line. Collectively, this study demonstrates that developing recombinant CHO cell lines to produce therapeutic proteins at high levels requires a balance of factors including transgene configuration, genomic locus landscape, and host cell properties.

## Graphical Abstract

**Figure:**
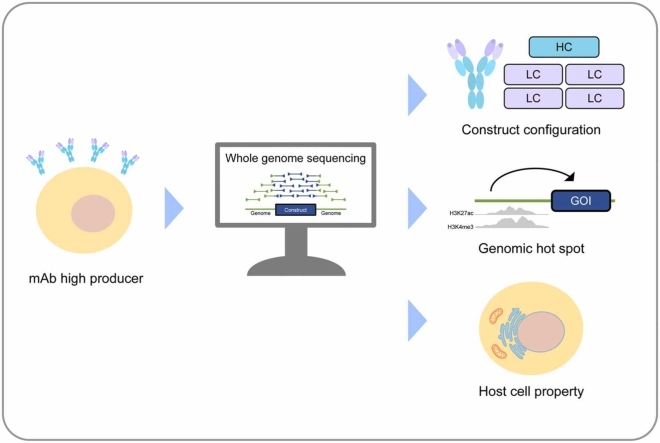

## 1 Introduction

Chinese hamster ovary (CHO) cells are a mammalian cell line widely used in the biopharmaceutical industry to produce therapeutic proteins. CHO cells have human-like post-translational modifications, low virus transmission, and can grow in serum-free media [1], [2], [3]. It also has a long FDA history, as it was the first approved mammalian cell line for biopharmaceutical production. Because of these advantages, as of June 2022, 211 of the 678 biopharmaceuticals approved in the United States and European Union have been produced using CHO cells [4].

To achieve high-yield production of therapeutic proteins in CHO cells, cell line development (CLD) is required. A common approach in CLD is the use of random integration. This method involves the random insertion of a therapeutic protein expression vector into the cellular genome, followed by the selection of cells showing robust protein expression and stability using antibiotics or metabolic selection processes [5]. In the industry, metabolic selection systems are preferred because antibiotics can act as contaminants during product purification [6]. Dihydrofolate reductase (DHFR)/methotrexate (MTX) and glutamine synthetase (GS)/methionine sulfoximine (MSX) are widely recognized metabolic selection systems [7]. Placing the gene of interest (GOI) with DHFR or GS in the expression vector allows transfected cells to be selected with their respective inhibitors MTX or MSX, respectively, while inducing amplification of copies of the GOI [8], [9]. However, when stable cell lines are established through random integration, each cell line has a different GOI integration site and copy number and epigenetic modifications can occur. This genomic plasticity leads to clonal variation [10]. To compensate for the limitations of random integration, a site-specific integration approach utilizes a clustered regularly interspaced short palindromic repeats (CRISPR)/CRISPR-associated protein 9 (Cas9) system to insert GOI expression cassettes at desired locations [11]. Site-specific integration mitigates clonal variation, leading to the development of stable and predictable recombinant cell lines [10]. To develop a cell line with a high production of therapeutic proteins through site-specific integration, a GOI expression cassette should be inserted into the ‘hot spot’, a chromosomal locus showing high transcriptional activity and stability. Hot spots are characterized by high chromatin accessibility and can exist within gene-annotated loci as well as in intergenic regions [12]. These sites can be identified by confirming and retargeting the integration sites using next-generation sequencing in cell lines with high expression levels of fluorescent proteins or therapeutic proteins generated through random integration. For example, the Fer1L4 site was identified by whole genome sequencing (WGS) in an antibody-producing cell line established by random integration and the hot spot was validated by FLP/FRT recombinase-mediated cassette exchange and CRISPR/Cas9-mediated homologous recombination [13], [14]. Hot spots can also be identified through PiggyBac and lentiviral screening, which integrate cargo into transcriptionally active chromatin regions, or by using safe harbors in mice or human cells, such as HPRT and Rosa26 [12], [15], [16], [17]. Although the significance of positional effects on therapeutic protein expression is evident, the mechanisms underlying these hot spots remain poorly understood [18].

Thus, we investigated the direct impact of genomic context on therapeutic protein expression in a hot spot of monoclonal antibody (mAb)-producing cell lines. We performed WGS to identify randomly inserted vectors in the genome of recombinant CHO (rCHO) cells that exhibited high mAb production, and experimentally validated these locations. To determine the impact of the transgene integration site on mAb production in rCHO cells, we analyzed the epigenetic features of the integration site and subsequently experimentally validated the site and sequence. Finally, we compared rCHO cells with host cells in terms of their inherent production capacity to provide insights into strategies for achieving high-yield production of therapeutic proteins.

## 2 Materials and methods

### 2.1 Plasmid construction

All sgRNA/Cas9 expression vectors and donor plasmids used in this study are shown in Suppl. Table 1. Single guide RNAs were designed using CRISPOR (version 5.01; [19]) and Cas-OFFinder [20], and cloned into the pU6-(*Bbs*I)_CBh-Cas9-T2A-mCherry (Addgene plasmid #64324; [21]) or pU6-(*Bbs*I)_CBh-Cas9-P2A-HygR (Addgene plasmid #127763; [22]). The duplex of single-stranded sgRNA was ligated into *Bbs*I (R0539L, New England Biolabs, Ipswich, MA, USA) digested #64324 or #127763 using T4 ligase (M0202L, New England Biolabs) ligation. Donor plasmids, including homology-directed repair (HDR)-mediated KI donor plasmids, were constructed using the uracil-specific excision reagent (USER) cloning method [11]. All bricks of the plasmids were amplified using PCR primers containing a single deoxyuracil residue and the Phusion U Hot Start DNA Polymerase (F555L; Thermo Fisher Scientific, Waltham, MA, USA). For etanercept (ETN) and EGFP-targeted integration, 5’ and 3’ homology arms targeting N3 or C2 in the IS14 site were obtained from genomic DNA of the GS-KO CHO-K1 cell line. For promoter-trap-based targeted integration [23], 5’ and 3’ homology arm targeting site 1 were obtained from promoter-trap CHO-K1 monitoring cell line genomic DNA. The PCR amplicon products were purified from 1% or 2% agarose Tris-Acetate-EDTA (TAE) gel with NucleoSpin® Gel and PCR Cleanup Kit (740609.250, Macherey-Nagel, Duren, Germany). All bricks of the USER cloning plasmid were assembled into the pcDNA3.1 backbone (Thermo Fisher Scientific) using the USER enzyme (M5505L, New England Biolabs). After T4 and USER cloning, the materials were transformed into *Escherichia coli* One Shot® Mach1™ competent cells (C869601; Life Technologies, Thermo Scientific, Rockford, IL, USA). All plasmids were purified using NucleoBond Xtra Midi EF (740420.50; Macherey-Nagel) and verified by Sanger sequencing. The primers used for all the plasmids are listed in Suppl. Table 2.

### 2.2 Whole genome sequencing (WGS)

Genomic DNAs were extracted from GS-knockout CHO-K1 host (GS-KO CHO-K1 WT) and GS-KO mAb cell lines. Then, WGS libraries were constructed using Illumina’s TruSeq DNA PCR-Free kit. After paired-end sequencing, the reads were aligned onto Chinese hamster assembly genome (RefSeq Accession: GCF_003668045.3) and mAb-expressing construct (Construct) using Burrows-Wheeler Alignment tool (BWA) (v0.7.17) [24]. To find Construct-integrated site on CHO contigs, paired-reads (e.g. R1 and R2) were separately aligned to Construct. Then, the reads were extracted when one-end of paired-read (e.g. R1) was aligned to Construct. After that, the other end (e.g. R2) was mapped to both references of CHO genome and Construct. Those paired-reads were further characterized as informative reads considering where the reads were mapped.

### 2.3 De novo assembly

To extend the upstream of Construct, the reads located at the forward region on Construct were assembled using Velvet assembler (v1.2.10) (e.g. contig A) [25]. After WGS reads were mapped to contig A, the mapped-reads on the forward 200 bp region of contig A were extracted and re-assembled to make another new contig (e.g. contig B). After 12 cycles of serial assembly and re-mapping, Construct integration site was characterized up to 5 kb upstream. At last, *de novo* assembled genome was mapped to CHO at the genome-wide level by BLAST (Basic Local Alignment Search Tool) [26].

For downstream of Construct, the reads that mapped on the last 1 kb of Construct were extracted and assembled to make a contig (e.g. contig C). Then, WGS reads were mapped to the contig C and re-assembled the reads to make new contig D, which is extended toward the downstream direction of contig C. While iterating this procedure, *de novo* assembled contig was revealed as parts of the middle region of Construct.

### 2.4 DeepSTARR

DeepSTARR is a deep learning model that directly predicts the activity of developmental and housekeeping enhancers in the DNA sequences of *Drosophila melanogaster* S2 cells [27]. To utilize DeepSTARR, the *de novo* assembled upstream region of Construct was binned into 249 bp windows with a stride of 1 bp. Then, one-hot encoded DNA sequences were used as input data for DeppSTARR to calculate developmental enhancer activity scores. The scores were assigned to genomic coordinates, then mean, minimum and maximum values per base were calculated. This tool is based on *D. melanogaster* S2 cells; therefore, it was used only as a reference in this study.

### 2.5 Cell lines and culture maintenance

The GS-KO CHO-K1 and GS-KO mAb cell lines were generated in the previous study [28]. Briefly, the GS-KO CHO-K1 cell line was established by using transcription activator-like effect nuclease technology to knockout the GS gene in CHO-K1 cell line. The GS-KO mAb clone was obtained by limiting dilution from a pool in which vectors containing the GS and mAb genes were randomly integrated into the GS-KO CHO-K1 host cell line. We used the KO-0 2–2 clone from Noh et al. [28]. The GS-KO CHO-K1 cell line was maintained in PowerCHO-2CD medium (BELN12–771Q, Lonza, Basel, Switzerland) supplemented with 8 mM L-glutamine (SH3003402, Hyclone, Logan, UT, USA). GS-KO mAb cell line was maintained in PowerCHO-2CD medium supplemented with GS expression medium supplement (GSEM, G9785, Sigma-Aldrich, St. Louis, MO, USA). Both cell lines were cultivated in 125 mL Erlenmeyer flasks (431143, Corning, Corning, NY, USA) with a working volume of 20 mL. All cell lines were incubated at 37 °C in a humidified 5% CO_2_ atmosphere. Viable cell density (VCD) and viability were measured using the trypan blue dye exclusion method with an automated cell counter (Countess II FL, Invitrogen, Carlsbad, CA, USA).

### 2.6 Generation and validation of indel mutation cell pools

A total of 1.0 × 10^6^ cells of GS-KO mAb were transfected with 10 μg of each sgRNA-Cas9-HygR plasmid for indel mutation using a NEPA21 electroporator (Nepagene, Chiba, Japan), as described previously [29]. After 2 d, transfected cells were seeded at 0.3 × 10^6^ cells/mL in suspension 6-well cell culture plate (351146, Falcon, Corning) with 3 mL PowerCHO-2CD medium supplemented with GSEM and 200 μg/mL hygromycin. Hygromycin selection was performed for the first 3 d, after which the medium was replaced with fresh medium and passaged every 3 d. Indel mutations were confirmed by Tracking of Indels by Decomposition (TIDE) analysis [27].

### 2.7 Generation of EGFP RI pools

To generate EGFP RI pools, a total of 1.0 × 10^6^ cells of GS-KO CHO-K1 or GS-KO mAb were transfected with 10 μg of each EGFP random integration donor plasmid using a NEPA21 electroporator. After 3 d, transfected cells were seeded at 0.5 × 10^6^ cells/mL in 125 mL Erlenmeyer flasks with 20 mL PowerCHO-2CD medium supplemented with 8 mM L-glutamine and 10 μg/mL puromycin dihydrochloride (P9620, Sigma Aldrich) or PowerCHO-2CD medium supplemented with GSEM and 10 μg/mL puromycin dihydrochloride. Cell pools were generated by puromycin selection for 2 weeks.

### 2.8 Generation of promoter KI monitoring cell line

The promoter-trap CHO-K1 monitoring cell line generated in a previous study contained a promoter-less EGFP expression cassette [23]. A total of 1.0 × 10^6^ cells of monitoring cell lines were transfected with 5 μg of sgRNA1 sgRNA-Cas9-mCherry and 5 μg of each promoter HDR KI donor plasmid using a NEPA21 electroporator for site-specific targeted integration. After 3 d, transfected cells were seeded at 0.3 × 10^6^ cells/mL in suspension 6-well cell culture plate with 3 mL PowerCHO-2CD medium supplemented with 8 mM L-glutamine and 500 μg/mL G418 disulfate salt solution (G8168, Sigma Aldrich). EGFP expression levels were determined using flow cytometry 6 d after transfection.

### 2.9 Generation and validation of ETN KI clone

To generate ETN KI clones, a total of 1.0 × 10^6^ cells of GS-KO CHO-K1 were transfected with 5 μg of each sgRNA-Cas9-mCherry plasmid and 5 μg of each ETN HDR KI donor plasmid using a NEPA21 electroporator for site-specific targeted integration. After 3 d, transfected cells were seeded at 0.1 × 10^6^ cells/mL in 125 mL Erlenmeyer flasks with 30 mL PowerCHO-2CD medium supplemented with 8 mM L-glutamine and 200 μg/mL zeocin (R25001, Thermo Fisher Scientific). Cell pools were generated by zeocin selection for 3 weeks, followed by limiting dilution. After that, the 5’/3’-juction PCR was performed to confirm the KI. The genomic DNAs of the clones in suspension 96-well cell culture plate (351172, Falcon, Corning) were extracted by QuickExtract™ (QE09050, Lucigen, Teddington, UK). The 5’/3’-juction PCR was performed using the Platinum™ SuperFi II PCR Master Mix (12368010, Invitrogen) by manufacturer’s PCR method (98 °C for 30 s; 30 ×: 98 °C for 10 s, 60 °C for 10 s, 72 °C for 40 s; 72 °C for 5 min). The 5’/3’-juction PCR primers used are listed in Suppl. Table 2.

### 2.10 Generation and validation of EGFP KI clone

A total of 1.0 × 10^6^ cells of GS-KO mAb were transfected with 5 μg of N3 sgRNA-Cas9-mCherry plasmid and 5 μg of N3 EGFP HDR KI donor plasmid using a NEPA21 electroporator for site-specific targeted integration. After 3 d, transfected cells were seeded at 0.5 × 10^6^ cells/mL in 125 mL Erlenmeyer flasks with 20 mL PowerCHO-2CD medium supplemented with GSEM and 5 μg/mL puromycin dihydrochloride. Cell pools were generated by puromycin selection for two weeks. EGFP positive and TagBFP negative clones were sorted using a FACS single-cell sorter (BD FACSAria Fusion; Becton Biosciences, Franklin Lakes, NJ, USA). After that, the 5’/3’-juction PCR was performed to confirm the KI. The genomic DNAs of the clones in suspension 96-well cell culture plates were extracted using QuickExtract™. The 5’/3’-juction PCR was performed using the Platinum™ SuperFi II PCR Master Mix by manufacturer’s PCR method. The 5’/3’-juction PCR primers used are listed in Suppl. Table 2.

### 2.11 Preparation of genomic DNA

Genomic DNA was extracted from 2.0 × 10^6^ cell pellets using the GeneJET Genomic DNA Purification Kit (K0721, Thermo Fisher Scientific) according to the manufacturer’s instructions. A NanoDrop 2000 spectrophotometer (Thermo Fisher Scientific) was used to measure the purified genomic DNA.

### 2.12 Tracking of Indels by DEcomposition (TIDE) analysis

The genomic DNA of the indel mutation and non-targeting cell pools was amplified using the Platinum™ SuperFi II PCR Master Mix. The PCR primers are designed in a way that the amplicons are ± 1000 bp, including the sgRNA target site and listed in Suppl. Table 2. The PCR amplicons were purified by NucleoSpin® Gel and PCR Cleanup Kit and verified using Sanger sequencing. TIDE online software (version 3.3.0; [30]) was used to calculate the indel frequency of CRISPR/Cas9. The parameter values were set to default values.

### 2.13 Batch culture

The GS-KO mAb cell line batch culture was seeded at 0.3 × 10^6^ cells/mL in 125 mL Erlenmeyer flasks with a working volume of 50 mL, and indel mutation and non-targeting cell pool batch cultures were seeded at 0.5 × 10^6^ cells/mL in 125 mL Erlenmeyer flasks with a working volume of 30 mL. A batch culture using ETN KI clones was seeded at 0.3 × 10^6^ cells/mL in a suspension 6-well cell culture plate with a working volume of 3 mL. VCD and viability were measured using a trypan blue dye exclusion assay with an automated cell counter. Culture supernatants were sampled and stored at − 70 °C. The antibody concentrations were measured using an enzyme-linked immunosorbent assay (ELISA) as previously described [31]. The specific mAb productivity (*q*_*mAb*_) was calculated using the integral viable cell concentration and mAb concentration (titer) in the exponential phase or on day 4.

### 2.14 Flow cytometry analysis to measure the levels of EGFP expression

All EGFP-expressing cells were analyzed using a NovoCyte Flow Cytometer (Agilent Technologies, Santa Clara, CA, USA), and the flow cytometry results were analyzed using NovoExpress software (Agilent Technologies) as previously described [32]. EGFP positive cells were gated using forward versus side scatter plots, and the fluorescence threshold was set to approximately 0.1% based on the EGFP negative control.

### 2.15 Quantitative real-time polymerase chain reaction

Genomic DNA from CHO-K1 WT, GS-KO CHO-K1, and GS-KO mAb cells was harvested on day 4 and purified using the GeneJET Genomic DNA Purification Kit. The relative target site copy number was analyzed using a CFX96 Real-Time System (Bio-Rad) with Power SYBR Green Master Mix (4367659; Applied Biosystems, Waltham, MA, USA). The number of target site was normalized to vinculin, and the obtained data was analyzed through the ΔΔC_T_ method.

### 2.16 Statistical Analysis

Results are presented as the mean ± standard deviation (SD) of three independent experiments. Statistical analysis was performed using an unpaired two-tailed *t*-test in GraphPad Prism 8 software (GraphPad Software, Inc., San Diego, CA, USA). Statistical significance was set at *P* < 0.05.

## 3 Results

### 3.1 Identification of construct integration sites

In a previous study, the mAb-producing rCHO cell line, GS-KO mAb (KO-0 2–2), was established through the random integration of a vector containing GS and mAb genes into the GS-knockout CHO-K1 host cell line [28]. The GS-KO mAb cell line showed specific mAb productivity (*q*_*mAb*_) above 20 pg/cell/day (pcd) after 30 passages of long-term culture [28] (Suppl. Fig. 1). Therefore, we hypothesized that the mAb gene could integrate into the genomic hot spot of the GS-KO mAb cell line.

**Fig. 1:**
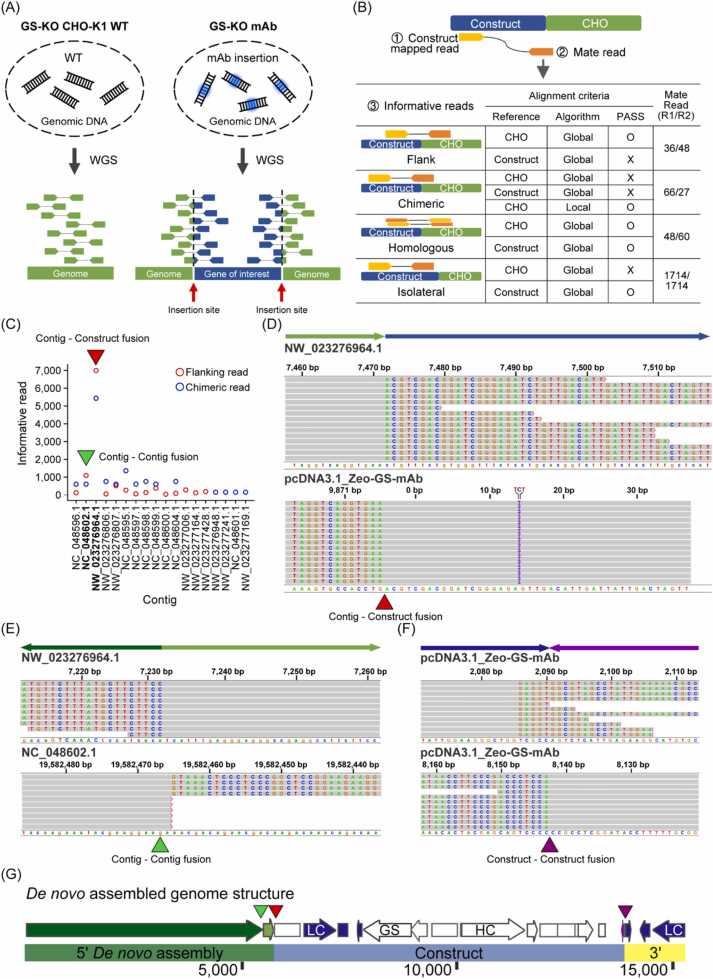
Discovering the construct integration site in the CHO genome. (A) Scheme of WGS analysis. (B) Definition of informative reads from paired-end reads (C) The number of flanking and chimeric reads in the CHO genome (D) IGV image of CHO contig (NW_023276964.1) and construct integration site [34]. Horizontal arrows represent direction of reference (light green, CHO; blue, construct). (E) IGV image of CHO contig (NW_023276964.1) - contig (NC_048602.1) fusion (light green, NW_023276964.1; dark green, NC_048602.1). (F) IGV image of Construct-Construct fusion. Horizontal arrows represent direction of reference . (G) *De novo* assembled genome structure where Construct was inserted. Triangle arrows represent fusion junctions and colors correspond to positions in Fig. 1C–F.

To identify the construct integration sites in the CHO genome, we performed WGS on GS-knockout CHO-K1 host (GS-KO CHO-K1 WT) and GS-KO mAb cell lines (Fig. 1A). Then, we aligned the paired-end reads to both the references of the CHO genome and the mAb-expressing construct (Construct). From the 1204,257,464 mapped reads, we defined and selected informative reads that were likely to encompass the junctional information of the integrated sites (Fig. 1B). Among the informative reads, the isolateral reads were mapped to the Construct, not the CHO genome (Suppl. Fig. 2A), whereas the other informative reads, such as flanking, chimeric, and homologous reads, were mapped to both the Construct and CHO genomes. As the Construct integrated is expected to span a few kilobases, the number of mate reads was high (1714) compared to other informative mate reads, which ranged 27–66. These reads were mapped on the narrow junctions between the Construct and CHO genome. In addition, the numbers of isolateral R1 and R2 reads were equal, suggesting properly paired reads on the Construct, whereas uneven numbers of other informative R1 and R2 reads indicates junctional mapping on two different contigs. Furthermore, because both the CHO genome and the Construct encode GS genes, the origins of homologous reads were ambiguous owing to sequence similarity (Suppl. Fig. 2B). Finally, using the flanking and chimeric reads, we determined the putative Construct-integrated CHO contigs (NW_023276964.1, NC_048602.1) (Fig. 1C, Suppl. Fig. 2C and D). To precisely identify the integration site at a single nucleotide resolution, we extracted non-aligned sequences from chimeric reads (soft clipped sequences from CIGAR string) by local alignment [33], re-aligned them to the CHO genome, and Construct (Suppl. Fig. 3). Surprisingly, fusion junctions were discovered between the CHO contig (NW_023276964.1) and Construct, as well as between the CHO contig (NW_023276964.1) and CHO contig (NC_048602.1) in GS-KO mAb cells (Fig. 1D and E).

**Fig. 2:**
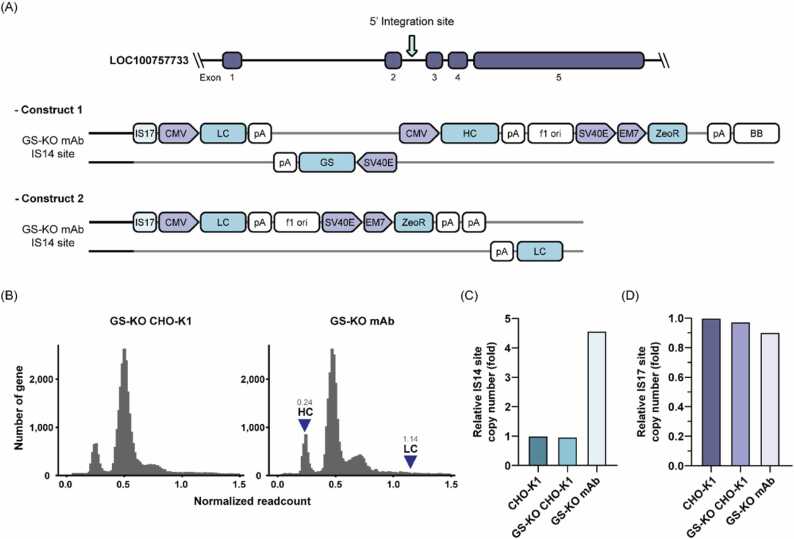
Comprehensive analysis and characterization of transgene integration site and construct. (A) Schematic illustration of the IS14 site (LOC100757733) and transgene construct. CHO genome sequence is represented by the black line; transgene sequence is represented by the gray line. Construct was assembled using Sanger sequencing. (B) Distribution of the sequencing depth in GS-KO CHO-K1 and GS-KO mAb cells. Arrows indicate the location of the HC and LC genes in the GS-KO mAb genome. (C) Relative copy number of the IS14 site in CHO-K1, GS-KO CHO-K1, and GS-KO mAb cells. (D) Relative copy number of the IS17 site (LOC113838965) in CHO-K1, GS-KO CHO-K1, and GS-KO mAb cells.

**Fig. 3:**
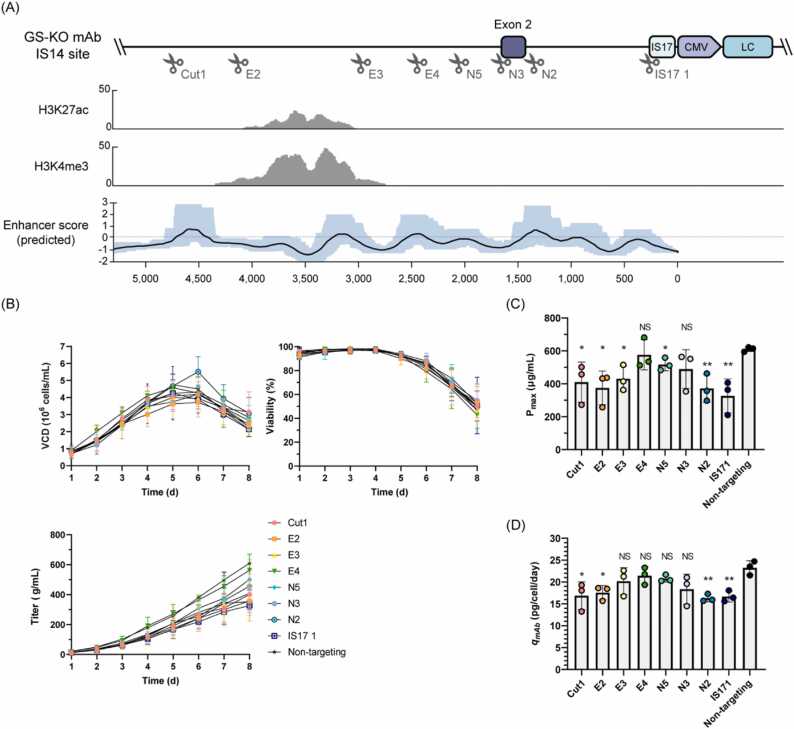
Generation and evaluation of the IS14 site indel mutation cell lines. (A) Illustration of eight sgRNA target sites, denoted as Cut1, E2, E3, E4, N5, N3, N2, and IS17 1, in the GS-KO mAb IS14 site with Genome Browser (GB) tracks of ChIP-seq signals and DeepSTARR enhancer predicted scores [40]. The Y-axis represents the normalized read counts and the X-axis shows the base pair distance from the CMV promoter. In the enhancer predicted score, the black solid line indicates the mean, and the blue background represents the maximum and minimum predicted scores. (B) Profiles of VCD, viability, mAb concentration (titer), (C) maximum product titer (P_*max*_), and specific mAb productivity (*q*_*mAb*_) of sgRNA-targeting cells and non-targeting cells during batch culture. Error bars represent the SD of three independent experiments. To determine the significance of the differences in means, an unpaired two-tailed *t*-test was used with the non-targeting pool as the control. * *P* < 0.05, ** *P* < 0.01; NS, not significant.

Although the local junctions were resolved, short reads were limited in revealing the distal upstream region because of the long genomic length of the CHO contig-contig fusion and the partial coverage of WGS reads upstream of the integration site (Suppl. Fig. 4A). Furthermore, the downstream region of Construct also contained a read-free locus, which was partially deleted during integration (Suppl. Fig. 4B). To investigate the distal upstream and downstream regions of Construct, we performed *de novo* assembly by genome walking (Suppl. Fig. 5). Briefly, Construct-mapped reads were *de novo* assembled, then WGS reads were re-mapped onto the assembled contig. The ends of the contigs were extended by assembling the re-mapped reads, including paired-end reads with only one mate mapped. Serial assembly and re-mapping cycles continued to extend the contig length. Finally, the *de novo* assembled contig was mapped to the CHO genome by Basic Local Alignment Search Tool (BLAST) [26]. As a result, Construct integration site was characterized up to 5 kb upstream, and the insertion of partial NW_023276964.1 and NC_048602.1 was also detected (Fig. 1. E, G and Suppl. Fig. 6). Moreover, the downstream structure showed multiple copies of partial Construct loci but not intact full-length integration (Fig. 1F and G). Collectively, these results indicate that the structure of the CHO genome and integrated construct is at high genomic instability in GS-KO mAb cells, and that *de novo* assembly is necessary to construct the genome structure of engineered CHO cells.

**Fig. 4:**
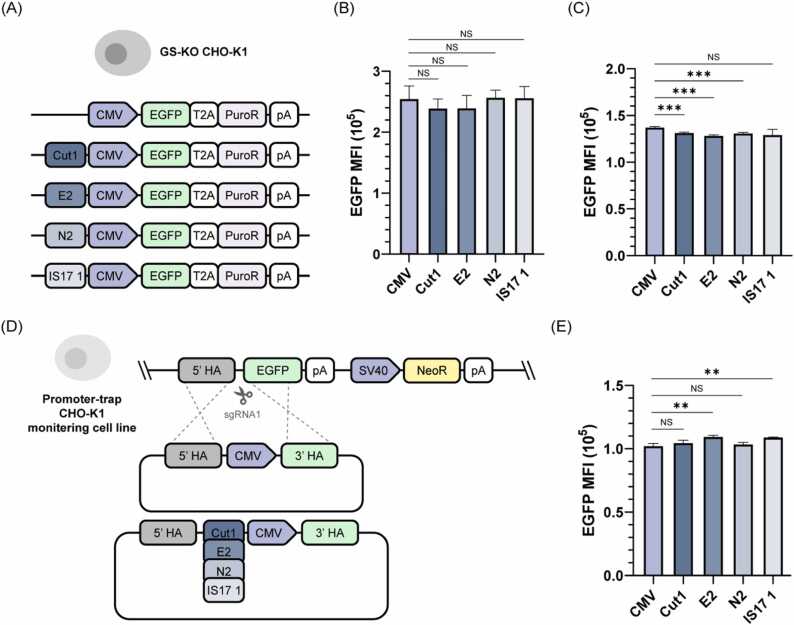
Effect of IS14 sequences on EGFP transient and stable expression. (A) EGFP expression vectors used for random integration into GS-KO CHO-K1 cells. MFI of EGFP positive populations during (B) transient expression and (C) stable expression generated by random integration. (D) Schematic illustration of HDR-mediated knock-in in the promoter-trap CHO-K1 monitoring cell line. The monitoring cell line has a promoterless EGFP expression cassette. (E) MFI of EGFP positive populations generated through promoter knock-in. Error bars represent SD of three independent experiments. To determine the significance of the difference in means, an unpaired two-tailed *t*-test was used. ** *P* < 0.01, *** *P* < 0.001; NS, not significant.

**Fig. 5:**
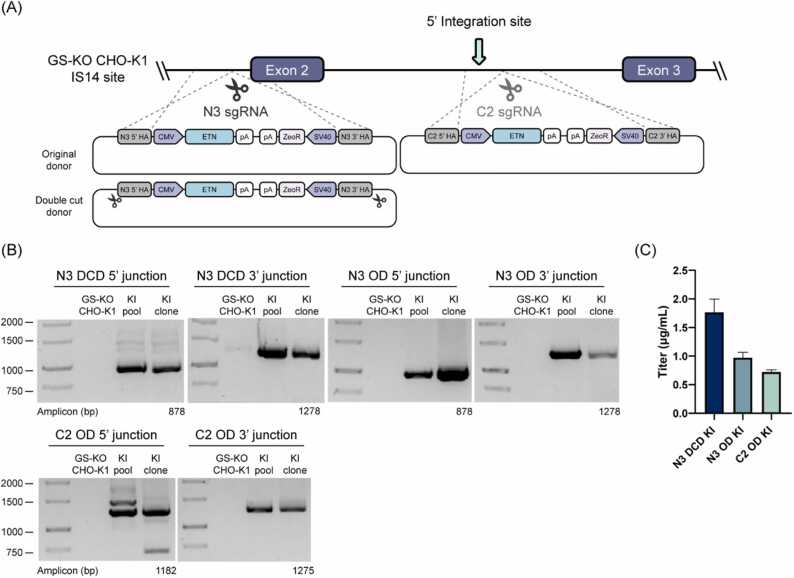
HDR-mediated KI into the IS14 site in the GS-KO CHO-K1 host cell line. (A) Schematic illustration of the site-specific integration of ETN expression cassette at the GS-KO CHO-K1 IS14 site. The OD is a conventional circular donor and the DCD is a modified version with a N3 sgRNA target site on both sides of the 5’ and 3’ homology arms. (B) 5’/3’-junction PCR analysis of ETN KI cell lines. The N3 DCD KI, N3 OD KI, and C2 OD KI pools were used as positive controls. (C) ETN protein concentration (titer) of three ETN KI clones. Error bars represent the SD of three independent experiments.

**Fig. 6:**
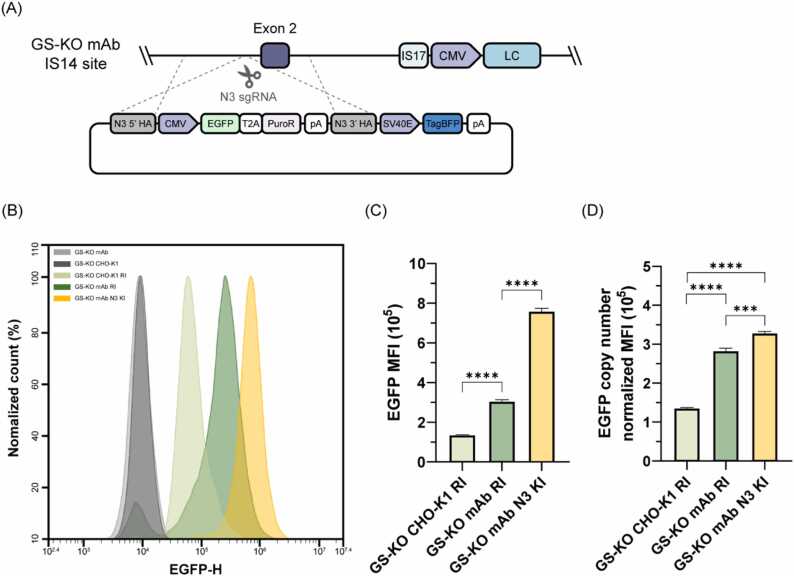
Evaluation of expression capacity of the IS14 site in GS-KO mAb cells. (A) Schematic illustration of HDR-mediated EGFP KI in the GS-KO mAb IS14 site. (B) Flow cytometry analysis of control (GS-KO CHO-K1, GS-KO mAb), GS-KO CHO-K1 EGFP RI pool, GS-KO mAb EGFP RI pool, and GS-KO mAb EGFP N3 KI clone. (C) MFI and (D) MFI normalized by EGFP copy number in EGFP positive papulations of GS-KO CHO-K1 EGFP RI pool, GS-KO mAb EGFP RI pool, and GS-KO mAb EGFP N3 KI clone. Error bars represent the SD of three independent experiments. To determine the significance of the difference in means, an unpaired two-tailed *t*-test was used, *** *P* < 0.001, **** *P* < 0.0001.

### 3.2 Construct integration site characterization

To confirm the 5’ construct integration site of *de novo* assembled genome structure in GS-KO mAb genome, we performed PCR and verified through Sanger sequencing (Suppl. Fig. 7). Sanger sequencing data were assembled to confirm the integration site and structure of the construct (Fig. 2A). The construct integration site is located between exons 2 and 3 of LOC100757733 and is hereafter referred to as IS14. LOC100757733 is a zinc finger protein 54 gene located on chromosome 9 of *C. griseus* [35]. The antibody expression cassette was inserted into the IS14 site in two distinct structures. Construct 1 was integrated into the intact form of a random integration vector, and Construct 2 was integrated into a modified form of a random integration vector without the GS and HC cassettes. Based on this result, the HC gene was found in one copy, and the LC gene was found in three copies in the GS-KO mAb. To confirm the copy numbers of the HC and LC genes in the WGS reads, we analyzed the distribution of the sequencing depth (Fig. 2B). GS-KO CHO-K1 genes and GS-KO mAb genes found in *C. griseus* genome exhibited similar distribution, showing distinct peaks at normalized readcount of 0.25, 0.5, and 0.75 corresponding to a copy number in the genome of one, two, and three copies. The sequencing depth analysis indicated that the HC gene was present in one copy and the LC gene in four copies of the GS-KO mAb genome. Therefore, HC and LC gene copy numbers exhibited similar patterns in PCR validation and distribution of sequencing depth.

Sanger sequencing revealed that the GS-KO mAb contained at least two IS14 sites. qRT-PCR was performed to determine the copy number of the IS14 site (Fig. 2C). We determined that the copy number of the genomic regions upstream of the IS14 site was approximately 4-fold higher in the GS-KO mAb than in CHO-K1 WT and GS-KO CHO-K1, indicating that chromosome-scale modifications occurred (Fig. 2C and Suppl. Fig. 8). Interestingly, we found that the 240 bp of NW_023276964.1 contig, hereafter referred to as the IS17, is located at the front of IS14 Constructs 1 and 2. IS17 is a flanking sequence originating from LOC113838965, a pseudogene with an uncertain chromosomal location. We assumed that the 3’ integration site was modified due to chromosome rearrangement between the IS14 site and the IS17 site, hence qRT-PCR was performed to confirm the copy number of the IS17 site (Fig. 2D). Following qRT-PCR analysis, we did not find any significant differences in the copy number of the IS17 site between CHO-K1 WT, GS-KO CHO-K1, and GS-KO mAb.

From these results, we verified the 5’ construct integration site and integrated transgene construct in the GS-KO mAb genome. In addition, it was evident that the integration site underwent significant chromosome-scale modifications during CLD through random integration (Suppl. Fig. 8).

### 3.3 Screening of the IS14 core locus

To determine the effect of IS14 on mAb productivity, we verified the features of the integration site. To investigate the epigenetic features of the upstream region at the integration site, we utilized chromHMM, an algorithm that identifies chromatin states by learning chromatin signatures based on a multivariate Hidden Markov Model [36], [37]. We collected six publicly available histone ChIP-seq datasets derived from studies on CHO cells (Suppl. Fig. 9A) [38], [39]. After learning the diverse chromatin signatures, we generated chromatin annotations consisting of 11 states. By applying chromatin annotation to the *de novo* assembled genome, we found that active histone marks (H3K27ac and H3K4me3) were enriched in upstream regions (Fig. 3A and Suppl. Fig. 9B). Therefore, we hypothesized that these marks increase antibody expression through elevated enhancer activity. To evaluate potential enhancer activity in the upstream region, we utilized DeepSTARR, a deep learning model that quantitatively predicts enhancer activity from DNA sequences [27]. Enhancer activity scores were calculated at a single-base resolution by averaging the sliding window approach (Fig. 3A). These results suggest that the region upstream of the transgene integration site likely functions as an enhancer. Although both sets of data suggest that the IS14 site shows enhancer activity, the mRNA expression of the IS14-associated gene was not detected in CHO-K1 WT, GS-KO CHO-K1, or GS-KO mAb (data not shown).

To determine the effect of the IS14 site on mAb productivity, a non-targeting sgRNA and eight sgRNAs were designed for indel mutations upstream of the IS14 site in GS-KO mAb (Fig. 3A). The GS-KO mAb cells were transiently transfected with all-in-one CRISPR/Cas9 plasmids targeting each upstream region, including a non-targeting control. Two days after transfection, hygromycin selection was performed for 3 d to enrich the transfected cells (Suppl. Fig. 10). After the cells were recovered, TIDE analysis was performed to measure the indel frequency of each sgRNA (Suppl. Fig. 11). All sgRNA targeting cell pools showed indel frequency over 49.5–93.5%, compared to the non-targeting cells. To assess mAb expression levels, eight indel mutations and non-targeting cell pools were cultured for 8 d (Fig. 3B). The batch culture profiles showed similar growth patterns across the cell pools. Notably, the maximum product titer of some of the indel mutation cell pools, including the Cut1, E2, E3, N2, and IS17 1 cell pools, was significantly decreased by more than 29.37% to 46.45% compared with non-targeting cells (Fig. 3C). The *q*_*mAb*_ of Cut1 (16.7 pcd), E2 (17.5 pcd), N2 (16.3 pcd), and IS17 1 (16.7 pcd) cell pools were 0.72-, 0.76-, 0.70-, and 0.72-fold than those of the non-targeting cells (23.3 pcd), respectively (Fig. 3D). Collectively, these results indicate that the upstream region of the IS14 site, specifically the locations targeted by Cut1, E2, N2, and IS17 1 sgRNAs, affects antibody productivity in GS-KO mAb cells.

### 3.4 Assessing the effect of the IS14 sequence

We further evaluated whether the sequence located at the IS14 site, where antibody production was reduced due to indel mutations, could be utilized as an element to enhance the expression of transgenes outside their native context. The 500 bp sequence around the sgRNA targeting sites Cut1, E2, N2, and IS17 1 in Fig. 3A was inserted next to the CMV promoter, which was identical to the promoter used for antibody production in GS-KO mAb, in the EGFP expression vectors (Fig. 4A). Three days after transfection of GS-KO CHO-K1 cells, transient expression was assessed by flow cytometry analysis of the mean fluorescence intensity (MFI) of EGFP positive populations (Fig. 4B and Suppl. Fig. 12A). There was no significant difference between the IS14 sequence-CMV- and CMV-only promoter-driven EGFP expression levels. Then, EGFP-expressing stable cell lines were generated by random integration using puromycin selection. Adding IS14 sequences in front of the CMV promoter resulted in a slight decrease in the MFI of EGFP positive populations in the random integration pools compared to CMV alone (Fig. 4C and Suppl. Fig. 12B).

To examine the effects of IS14 sequences within a more consistent and homogeneous genomic context, we generated targeted integrants using a promoter-trap CHO-K1 monitoring cell line. The promoter-trap CHO-K1 monitoring cell line generated in a previous study contained a promoterless EGFP expression cassette [23] (Fig. 4D). The sgRNA1-Cas9 expression vector was co-transfected with HDR donors, which had IS14 sequences adjacent to the CMV promoter or the CMV promoter only, to restore EGFP expression [29]. Six days after transfection, the MFI of EGFP positive cells was examined (Fig. 4E and Suppl. Fig. 12C). The presence of E2 and IS17 1 sequence in front of the CMV promoter showed a significant increase compared to CMV only, but the absolute values were not dramatic, at 7.07% and 6.68% increases, respectively. Overall, the IS14 sequence, where antibody production was reduced owing to indel mutations in the GS-KO mAb, did not increase transgene expression levels in GS-KO CHO-K1 host cells, regardless of the expression mode.

### 3.5 Validation of the IS14 site effects in host cell line

To confirm that the IS14 site functions as a hotspot in host cells, we performed HDR-mediated knock-in (KI) using the CRISPR/Cas9 system at the IS14 site in the GS-KO CHO-K1 host cell line. We designed N3, C2 sgRNAs and homology arms at the front and behind the 5’ integration site in GS-KO CHO-K1 genome, respectively (Fig. 5A). The HDR KI donors were comprised of a 5’ and 3’ homology arm, an ETN expression cassette, and a zeocin resistance gene expression cassette. We expressed a single-chain protein, ETN, as the model protein to focus on the effect of the site itself, independent of the effect of the chain ratio and position of the HC and LC of the antibody on productivity. There are two types of N3 HDR KI donors: the original donor (OD) and double cut donor (DCD). DCD was used to increase KI efficiency and it has N3 sgRNA target site on both sides of the 5’ and 3’ homology arms [29]. Three days after transfection with N3 or C2 HDR KI donors and N3 or C2 sgRNA-Cas9 expression vectors, Zeocin selection was performed. To obtain KI clones, we performed limiting dilution of N3 or C2 ETN KI pools. Low numbers of 5’/3'-junction PCR-positive cells after limiting dilution led to the isolation of only one ETN KI clone each from N3 DCD, N3 OD, and C2 OD ETN KI pools (Fig. 5B and Suppl. Fig. 13). To assess ETN expression levels, N3 DCD, N3 OD, and C2 OD ETN KI clones were cultured in 6-well plates for four days. ETN expression was visible; however, absolute expression levels were low in all KI clones, averaging 1.15 µg/mL (Fig. 5C). The results demonstrated that the IS14 site in the host cell line did not possess the capacity for high-level expression of the therapeutic protein as a hot spot.

### 3.6 Validation of the IS14 site effects in GS-KO mAb cell line

As we did not confirm the IS14 site as a hot spot in the host cell line, we examined the effect of the IS14 site with modified 3' genomic contents on protein expression within its native context, GS-KO mAb cell line. GS-KO mAb cells express exogenous therapeutic proteins; therefore, we used EGFP as the protein of interest. We performed CRISPR/Cas9-mediated KI at the IS14 site in the GS-KO mAb cells (Fig. 6A). The HDR KI donors contained a 5’ and 3’ homology arm, an EGFP-T2A-puromycin resistance gene expression cassette, and a TagBFP expression cassette. As shown in Fig. 2A and C, the GS-KO mAb harbors more than one IS14 site, so we utilized TagBFP external to the 3’ homology arm to identify vector random integration. Three days after transfection with the EGFP HDR KI donor and the N3 sgRNA-Cas9 expression vector, puromycin selection was performed. EGFP positive and TagBFP negative cells were isolated using FACS after puromycin selection, followed by KI validation using 5’/3’-junction PCR (Suppl. Fig. 14). In parallel, we generated EGFP random integration (RI) pool cells from the GS-KO mAb cell line to compare EGFP expression levels derived from either the IS14 site or random genomic sites. A CMV-only EGFP expression vector (Fig. 4A) was used for RI. The MFI of the EGFP positive population was 2.49-fold higher for the GS-KO mAb N3 KI clone than for the GS-KO mAb RI pool (Fig. 6B, C and Suppl. Fig. 15). However, the GS-KO mAb N3 KI clone harbored approximately two copies of EGFP (Suppl. Fig. 16). Therefore, the normalized MFI of the EGFP positive populations, relative to the EGFP copy number, was slightly higher than that of the GS-KO mAb RI pool by 1.16-fold (Fig. 6D). This indicated that the transcriptional activity of the IS14 site was not superior to the average transcriptional activity of random genomic loci, although the region upstream of the IS14 site was involved in high mAb production. Interestingly, when comparing the MFI of the random pool established from the GS-KO CHO-K1 cells (Fig. 4C) with that of the GS-KO mAb RI pool, the GS-KO mAb RI pool displayed a 2.09-fold higher normalized MFI of EGFP positive populations than the GS-KO CHO-K1 RI pool, even though the cells were generated using the same EGFP expression vector. Collectively, these findings suggest that the IS14 site did not act as a hot spot in the parental GS-KO mAb cell line and that the exogenous protein expression capacity of the GS-KO mAb cell line was superior to that of its host cell line, GS-KO CHO-K1.

## 4 Discussion

Genomic hot spots continue to be identified based on the benefits of site-specific integration, such as reduced clonal variation and predictable phenotypes. In this study, we used WGS to identify transgene integration sites in rCHO cells established through random integration and experimentally validated the sites and cell characteristics.

The IS17 CHO contig (NW_023276964.1) - Construct and IS17 CHO contig (NW_023276964.1) - IS14 CHO contig (NC_048602.1) fusion junctions were observed in the GS-KO mAb, and *de novo* assembly was performed to identify the IS14 site as the 5' construct integration site (Fig. 1G and Suppl. Fig. 5). Thus, the IS17 contig, which was not originally present at the IS14 site, was placed in front of the transgene (Fig. 2A). Additionally, we checked the copy number of the IS14 site of the GS-KO mAb and confirmed that the copy number was higher than 4-fold compared of wild-type CHO-K1 (Fig. 2C). These results suggest that random insertion of expression vectors into the genome leads to genetic modifications, such as chromosomal rearrangement. Other studies have confirmed that transgene integration sites in cell lines established by random integration are modified by unique chromosomal rearrangements and are not implemented in host cells [41]. As reported, chromosomal rearrangement was evident in GS-KO CHO-K1 mAb, particularly near the IS14 site, based on the analysis of copy number variation (Suppl. Fig. 8). Functional analysis of genes with increased copy numbers in the GS-KO CHO-K1 mAb revealed that the amplified genes were significantly associated with nucleosome assembly (Suppl. Fig. 17). Because nucleosome assembly is a crucial mechanism for chromatin stability and gene regulation, epigenetic remodeling through genomic rearrangement may play a significant role in elevating mAb expression.

Although the expression vector used to establish the stable GS-KO mAb cell line contained one copy of the HC and LC expression cassettes, the distribution of sequencing depth identified one copy of HC and four or more copies of LC (Fig. 2B). PCR validation further supported that the LC cassettes were more than three copies, and one of the LC was inverted at 3’ in Construct 2 (Fig. 2A). Due to this configuration, the 3’ CHO contig was not found in the *de novo* assembled genome structure but the LC cassette instead (Fig. 1G). To overcome the limitations of analyzing these short reads, targeted gene amplification (TLA) or long-read sequencing can be used to identify transgene integration sites [42], [43]. Additionally, in the GS-KO mAb cell line, the copy number ratio of HC to LC was approximately 1:4 (Fig. 2B). Free HCs accumulate in the endoplasmic reticulum until they assemble with LCs, resulting in ER accumulation when free HCs are abundant [44]. Therefore, a higher LC ratio is more beneficial for antibody production as long as the basic expression of HC is ensured [45]. HC:LC peptide ratios ranging 1:2–1:3 were observed in stable high-mAb-producing pools [46], and LC to HC transgene copy number ratio ranging 2.2–3.5 was measured for high-producer clones [47]. Thus, high antibody productivity by the GS-KO mAb cell line was attributed to the optimal HC:LC ratio.

Characterization of the IS14 site revealed active histone marks and locations with high enhancer prediction scores (Fig. 3A). To determine whether such locations at the IS14 site affect antibody productivity, we generated indel mutations using CRISPR/Cas9 in the IS14 site of the GS-KO mAb. Examination of the maximum product titer and specific productivity revealed a significant decrease in productivity at the Cut1, E2, N2, and IS17 1 sgRNA target sites (Fig. 3C and D). The Cut1 and E2 sites were in front of the active histone marks, and the Cut1, E2, and N2 sgRNA target sites showed high DeepSTARR enhancer prediction scores, suggesting that changes in the genomic context of the sgRNA target sites due to indels led to reduced antibody production.

Previous studies demonstrated that chromosome-integrated reporter assays, which integrate a vector with a putative enhancer sequence in front of the reporter into the genome, reflect endogenous enhancer activity [48]. Therefore, we examined whether the sgRNA target IS14 sequence, which had a high DeepSTARR enhancer score and resulted in lower antibody productivity upon indel generation, affected the expression of exogenous proteins (Fig. 4A and D). However, no significant increase in EGFP expression was observed in either transient or stable expression compared to CMV alone (Fig. 4B, C, and E). Therefore, we conclude that these sgRNA target sequences have an effect within a complex genomic context.

To validate the IS14 site as a hot spot in host cells, we integrated a single-chain therapeutic protein, ETN, expression cassette, into a region with active histone marks and a high DeepSTARR enhancer prediction score; however, high levels of production were not observed (Fig. 5C). We also confirmed that the EGFP KI in the 3’ modified IS14 site of the GS-KO mAb cells was not acting as a hot spot to increase the expression level of EGFP, as the EGFP copy number-normalized MFI was slightly higher (1.16-fold) compared to the GS-KO mAb pool with randomly inserted EGFP (Fig. 6D). However, the modified IS14 copy number in GS-KO mAb cells has the advantage of allowing the insertion of more than one copy of the desired GOI with a single-targeting sgRNA. Interestingly, the pool with randomly inserted EGFP in GS-KO mAb cells had a 2.09-fold higher EGFP copy number-normalized MFI of EGFP positive populations than the pool with randomly inserted EGFP in host cells. This suggests that the GS-KO mAb cells were selected as high-producing cells and therefore have potentially robust protein expression capacities compared to the host cells.

## 5 Conclusion

In this study, we identified the transgene integration site and composition of the rCHO cell line producing mAb to investigate the effect of the genomic locus on high-level protein production. This cell line harbored multiple copies of LC due to the vector rearrangement upon random integration, and the genomic context was modified by chromosome rearrangements, particularly at the 3’ transgene integration site and CHO contig-contig fusions, which affected antibody productivity. In addition, compared to the host cells, the rCHO cell line exhibited a preferable protein production capacity. Collectively, the high level of therapeutic protein production in stable cell lines can be attributed to vector configuration such as copy number and structural rearrangement of vector elements in the genome, genomic context including genomic rearrangement and transgene integration site, and host cell capacity. As the modified genomic context and phenotypic changes that occur during the random integration of transgenes are challenging to implement in wild-type CHO cells, integration sites discovered through high producers should be used with care. To develop high producers while maintaining the advantages of site-specific integration, utilizing platform cell lines with high protein productivity capabilities and reusable integration sites, such as the recombinase-mediated cassette exchange-based targeted integration approach, could be an alternative CLD platform.

In conclusion, the present study provides insights into the multiple variables underlying high protein production in rCHO cells. It was found that rCHO cells generated through random integration were accompanied by modifications in the genomic context that affected interactions with the transgene and inherent properties of the cell line. Further characterization of interactions among structural variations of the rCHO genome, vector configuration, and functional phenotypic changes could uncover the underlying specific features of recombinant protein production and drive streamlined site-specific integration-based CLD.

## CRediT authorship contribution statement

**Dongwoo Kim:** Investigation, Methodology. **Jae Yun Moon:** Formal analysis, Methodology. **Daechan Park:** Conceptualization, Funding acquisition, Methodology, Supervision, Writing – review & editing. **Jae Seong Lee:** Conceptualization, Funding acquisition, Methodology, Supervision, Writing – review & editing. **Hyun Jee Woo:** Investigation, Methodology, Writing – original draft. **Jaehoon Kim:** Formal analysis, Investigation, Writing – original draft. **Seul Mi Kim:** Investigation, Methodology.

## Declaration of Competing Interest

The authors declare that they have no known competing financial interests or personal relationships that could have appeared to influence the work reported in this paper.
