# Supplementary material for: Context-dependent genomic locus effects on antibody production in recombinant Chinese hamster ovary cells generated through random integration
Source: Comput Struct Biotechnol J. 2024 Apr 10;23:1654–65. doi: 10.1016/j.csbj.2024.04.023 (PMC11046053; doi:10.1016/j.csbj.2024.04.023)
Supplement: Supplementary file 1 — Supplementary material [file mmc1.docx]

**Supplementary Data**


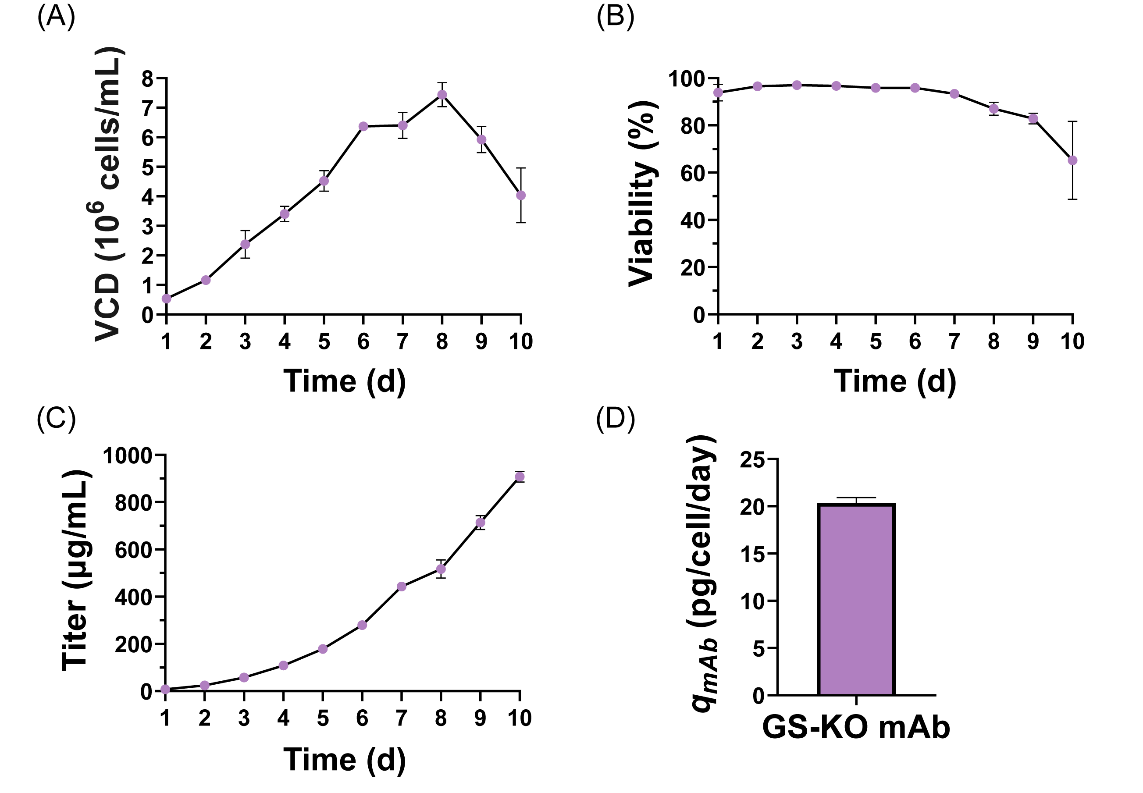


**Supplementary Figure 1.** Batch culture profiles of GS-KO mAb cell line. Profiles of (A) VCD, (B) viability, (C) mAb concentration (titer), and (D) specific mAb productivity (*q_mAb_*) of the GS-KO mAb cell line during batch culture. Error bars represent the SD of three independent experiments.


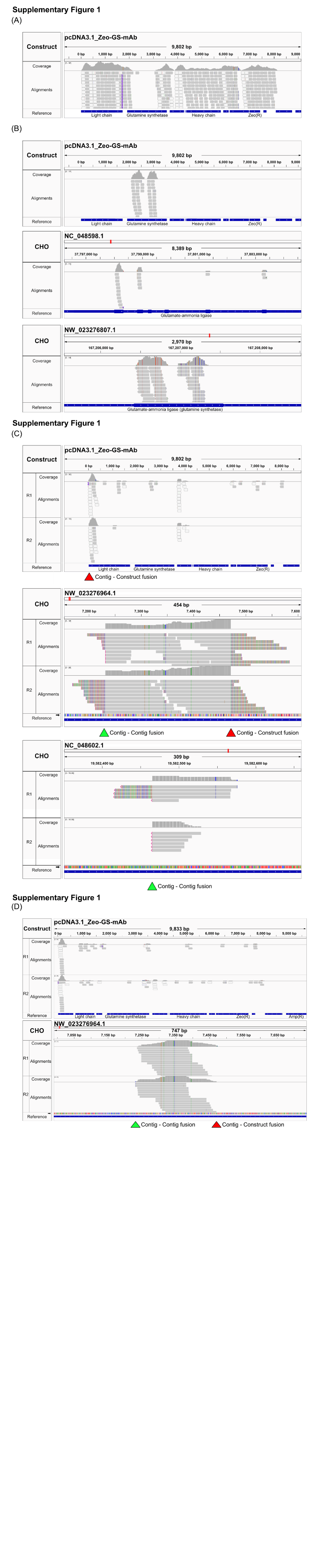


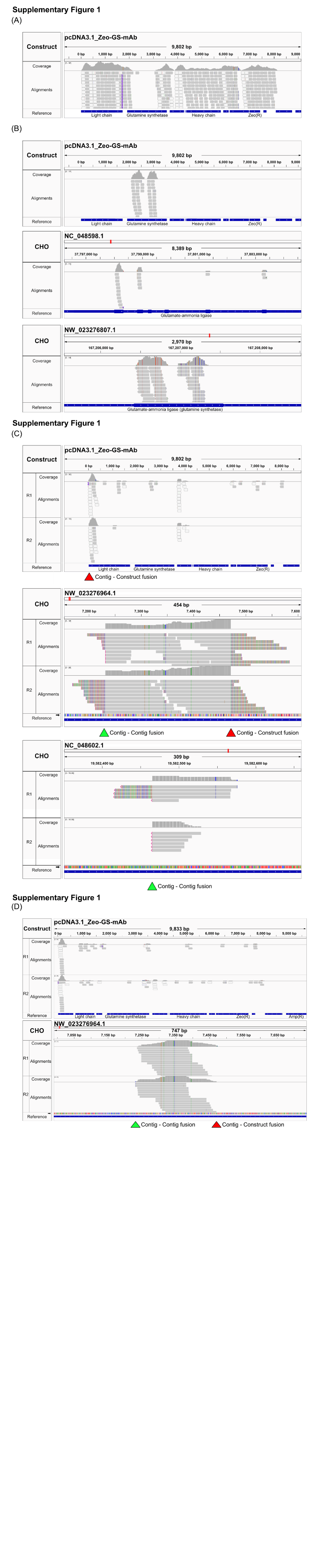


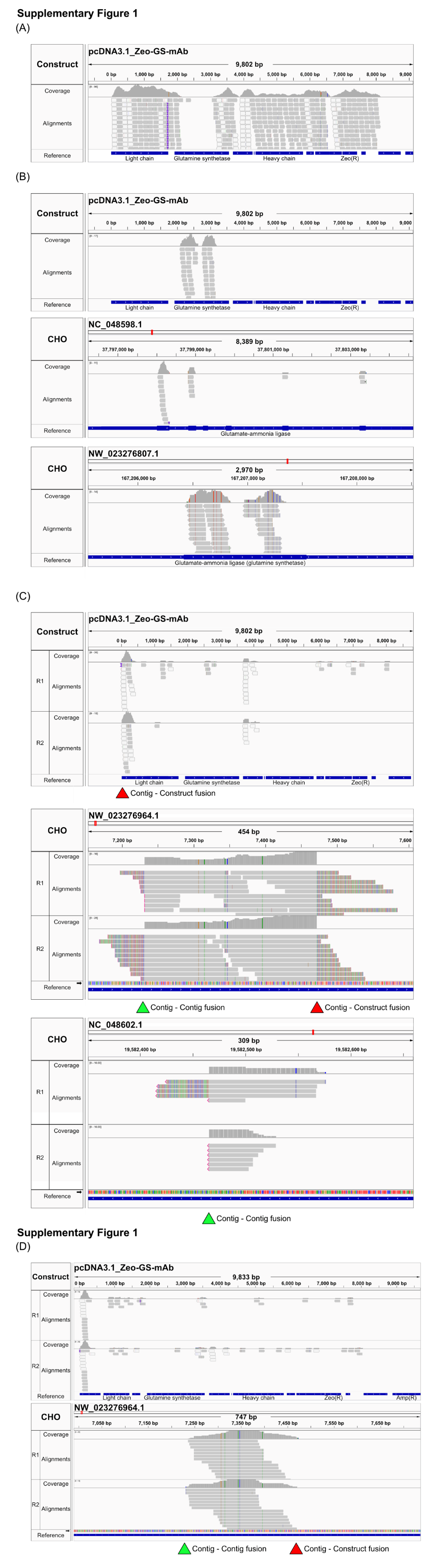


**Supplementary Figure 2.** IGV image of informative reads. Informative reads visualized at the CHO genome, Construct, and genomic fusion sites. (A) Isolateral reads are only mapped to the Construct (pcDNA3.1_Zeo-GS-mAb) (B) Pairs of homologous reads on Construct (top) are located at glutamate-ammonia ligase gene (Glul, ENSCGRG00015037572) (middle) and glutamate-ammonia ligase pseudogene (Glul pseudogene, ENSCGRG00015014735) (bottom). Most chimeric (C, top) and flanking (D, top) reads on the Construct are located at the forward region. Other pairs are aligned with the NW_023276964.1 contig, where the integration site is located (C, middle and D, bottom). CHO contig (NW_023276964.1) - contig (NC_048602.1) fusion is found by chimeric reads (C, bottom).


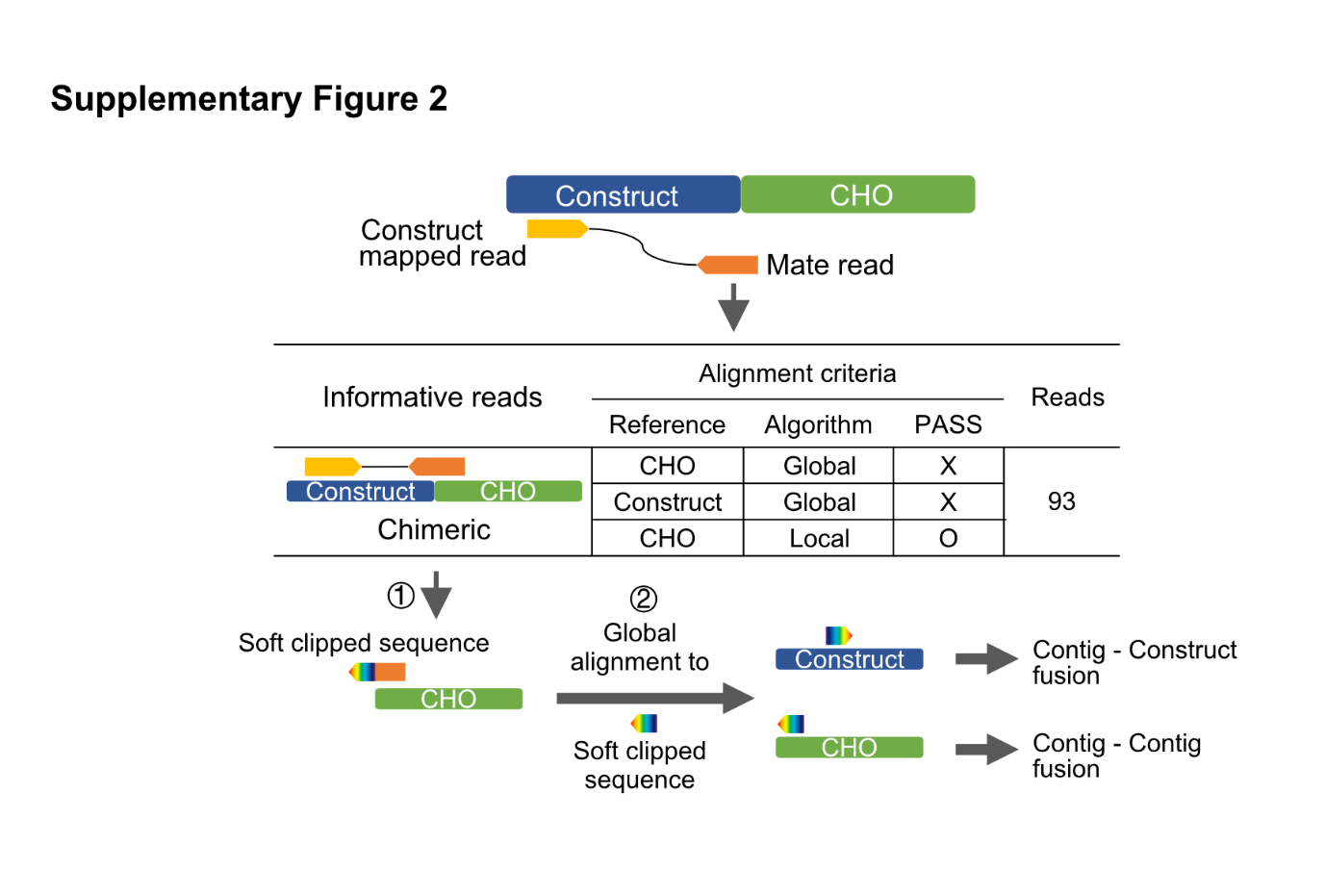


**Supplementary Figure 3.** Investigation of chimeric reads. First, chimeric reads partially aligned to CHO genome (e.g. NW_023276964.1 contig) were extracted. Next, unaligned sequences (soft clipped sequences) from partially aligned reads were re-mapped to the Construct and CHO genome. Finally, CHO contig-Construct and CHO contig-contig fusion were defined when the reads were re-mapped to Construct or a different CHO contig (e.g. NC_048602.1 contig).


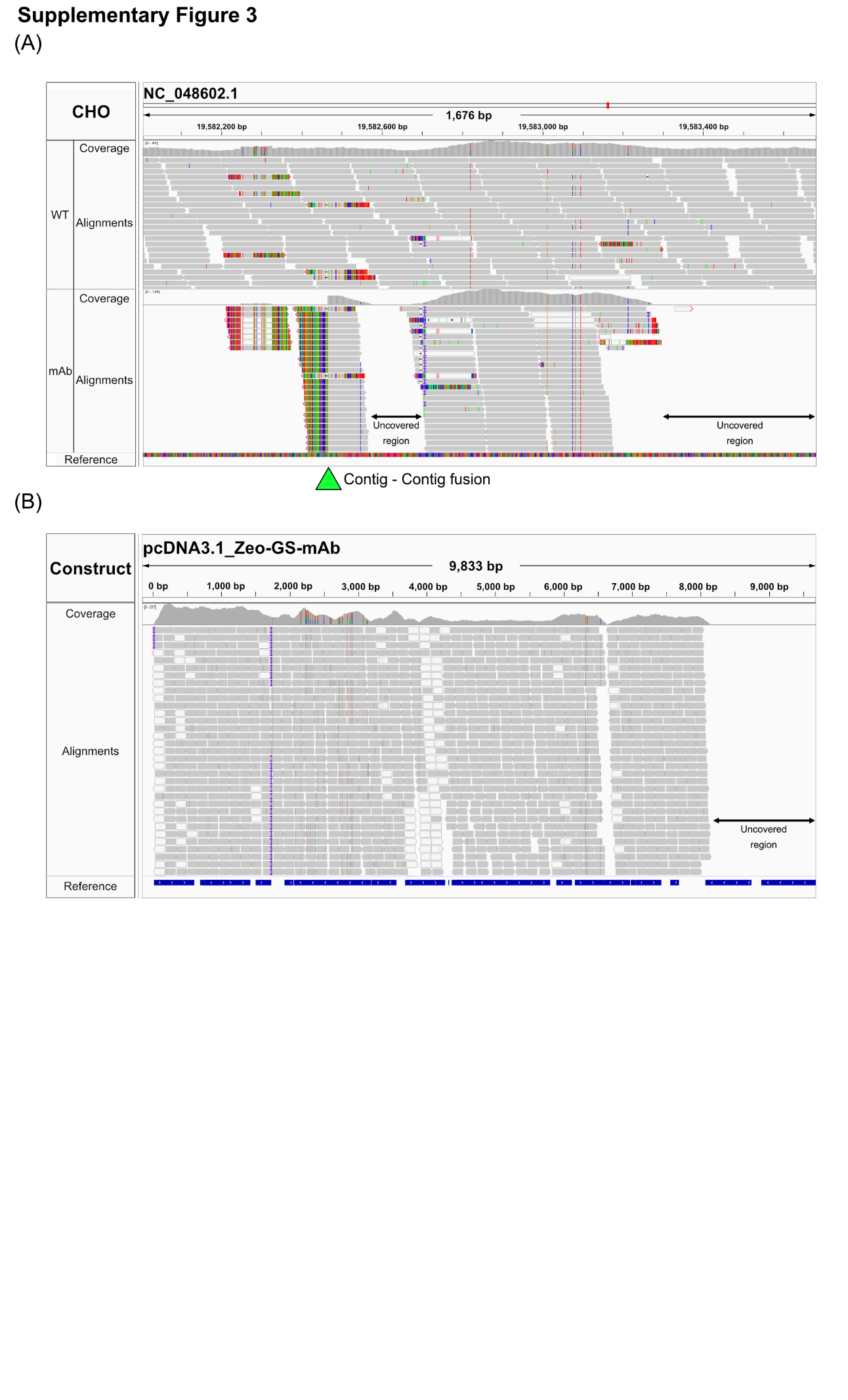


**Supplementary Figure 4.** IGV image of the uncovered region. (A) Upstream of the integration site and (B) Construct region not fully covered through WGS reads.


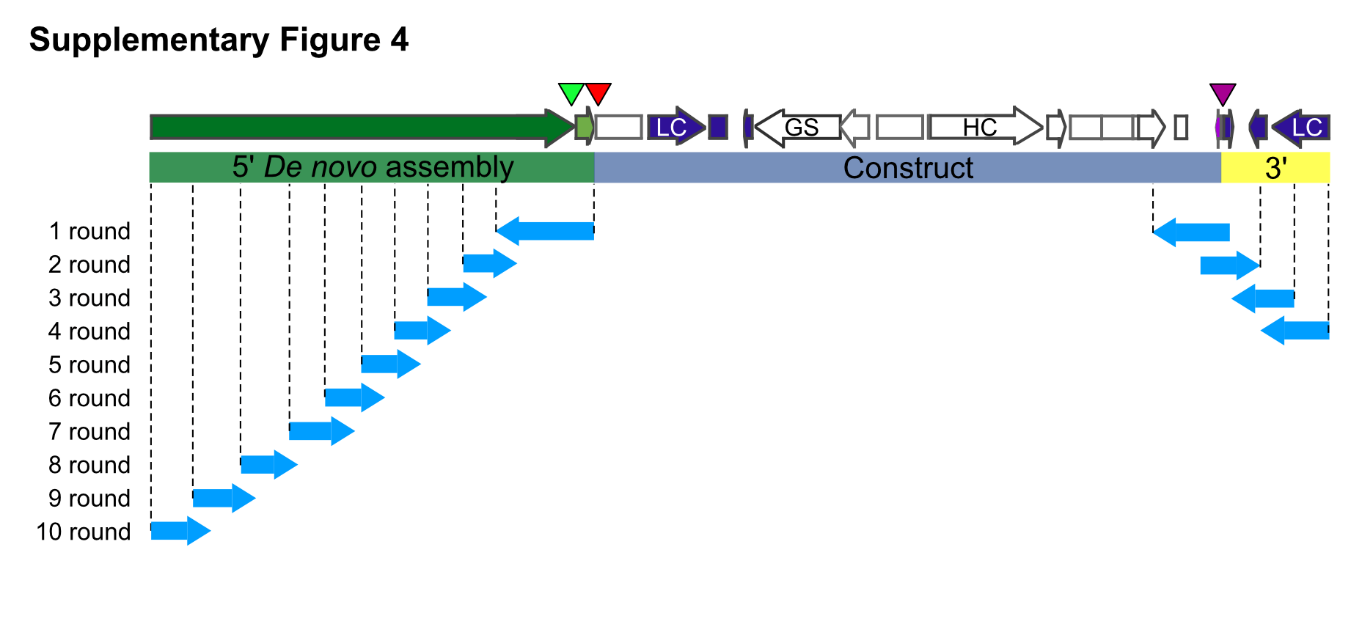


**Supplementary Figure 5.** *De novo* assembly of upstream and downstream of Construct. Upstream and downstream of Construct were assembled using serial assembly. Approximately 5 kb upstream of the integration site (red) was elucidated, and fusion structures such as CHO contig-contig fusion (green) were characterized. Downstream, multiple copies of partial Construct loci were elucidated using *de novo* assembly.


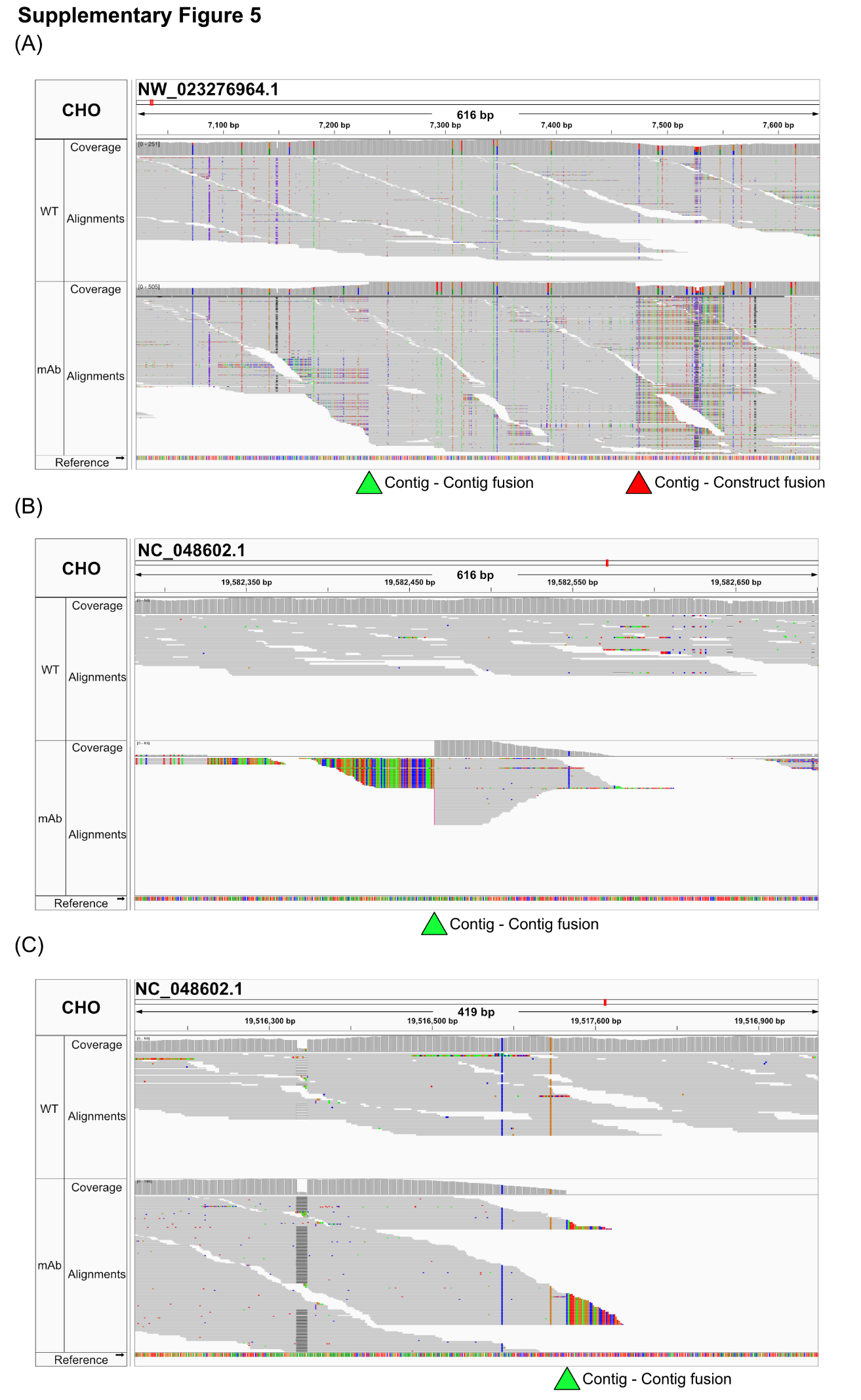


**Supplementary Figure 6.** IGV image of upstream of Construct in CHO genome reference. (A and B) CHO Contig–Construct (Construct integration site) fusion and CHO contig-contig fusion were identified by informative reads. (C) Using BLAST, CHO contig-contig fusion was re-mapped on CHO genome.


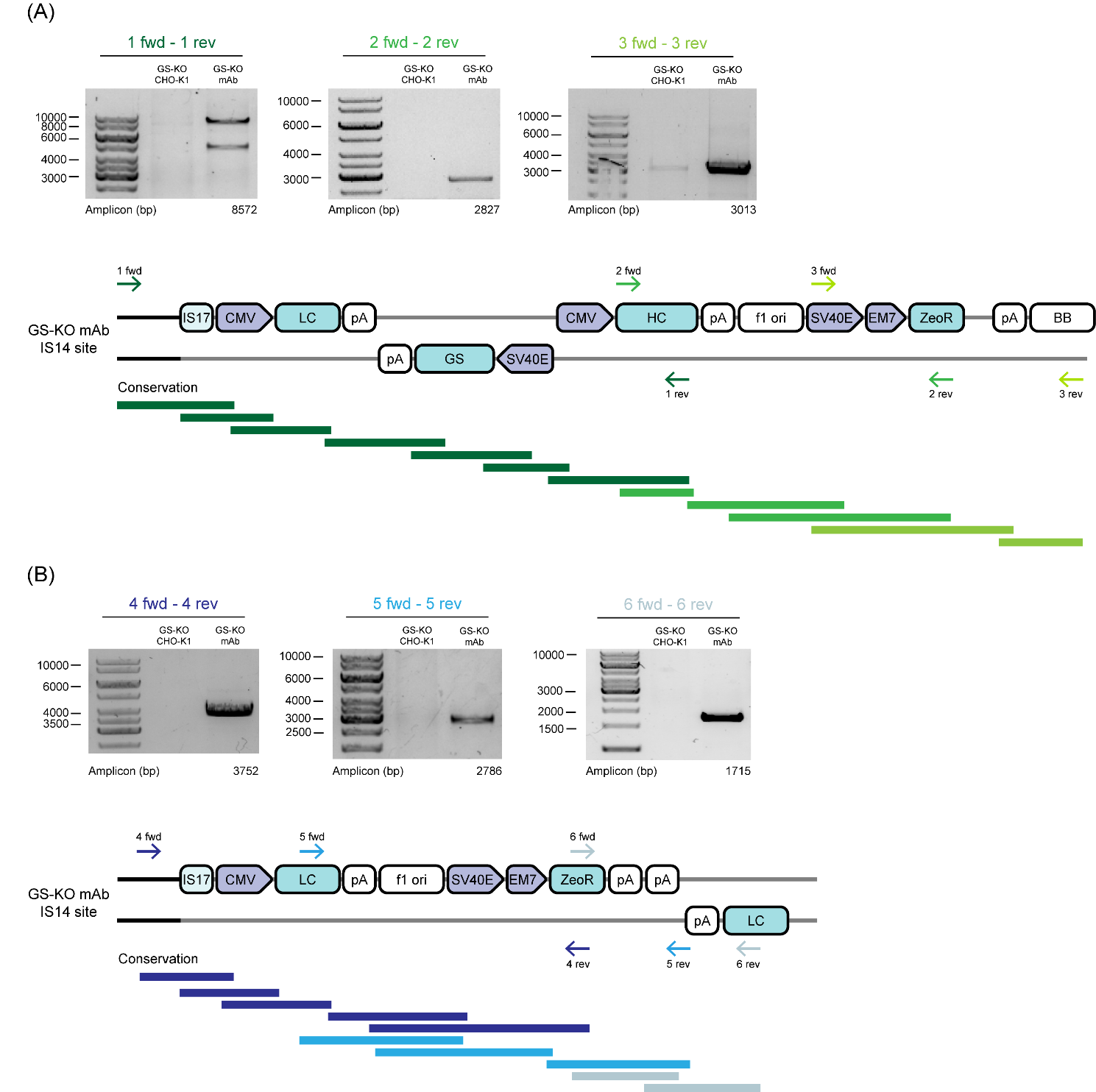


**Supplementary Figure 7.** Validation of the GS-KO mAb genome IS14 site using PCR analysis. (A) Constructs 1 and (B) 2 were validated using PCR analysis and verified using Sanger sequencing. PCR primers were designed to overlap to confirm merging. Primer sequences used for PCR analysis are listed in Suppl. Table 2. Sanger sequencing conservation blocks from a single amplicon are labeled with the same color.

**
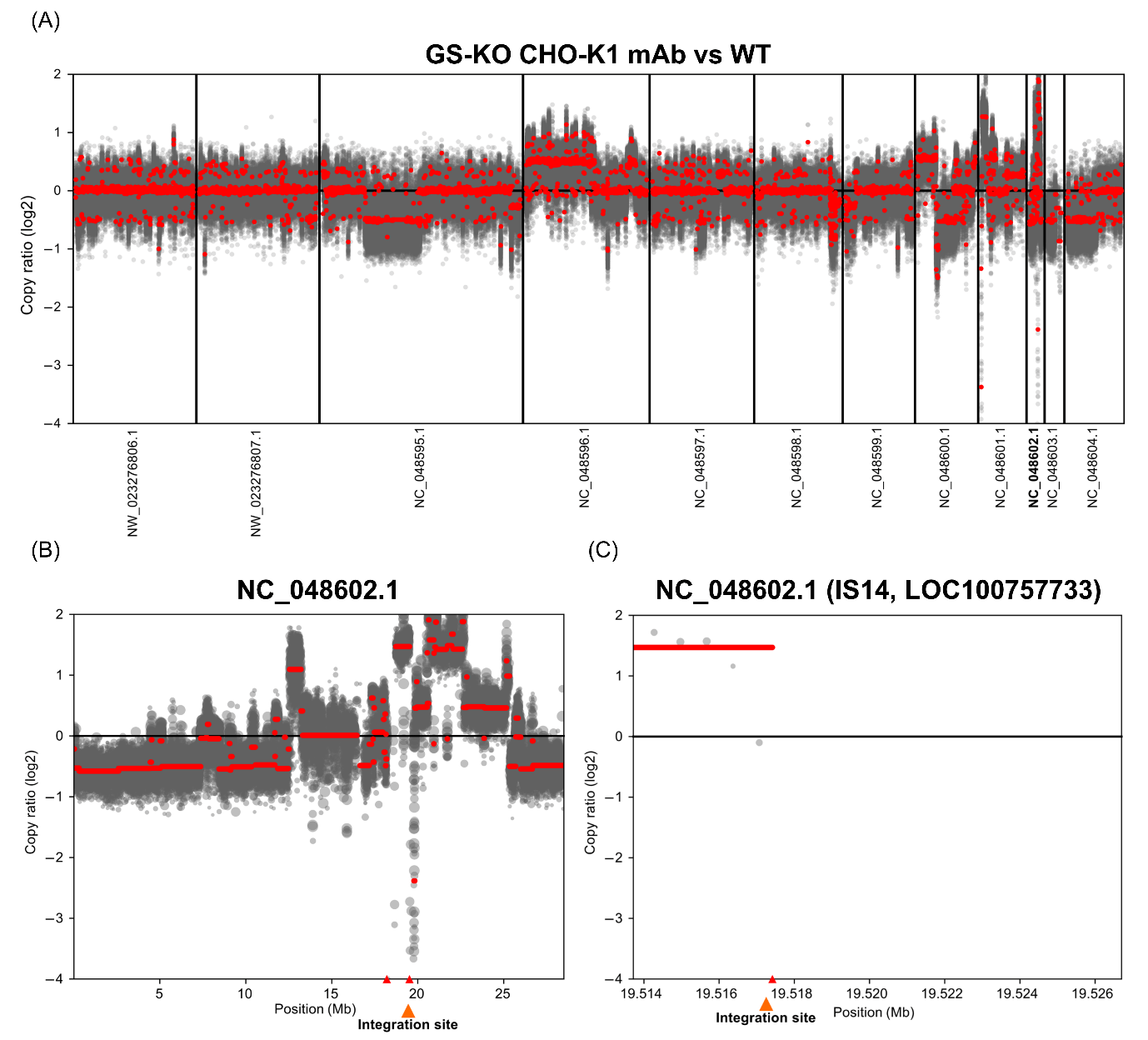
**

**Supplementary Figure 8.** Copy number variation (CNV) analysis. CNVkit was used to assess CNV between GS-KO CHO-K1 mAb and WT [1]. The copy number ratio was calculated using log2 (copy numbers of mAb/copy numbers of WT). The CNV profile was visualized for the whole genome (A), the contig, and the Construct integration site (B and C)


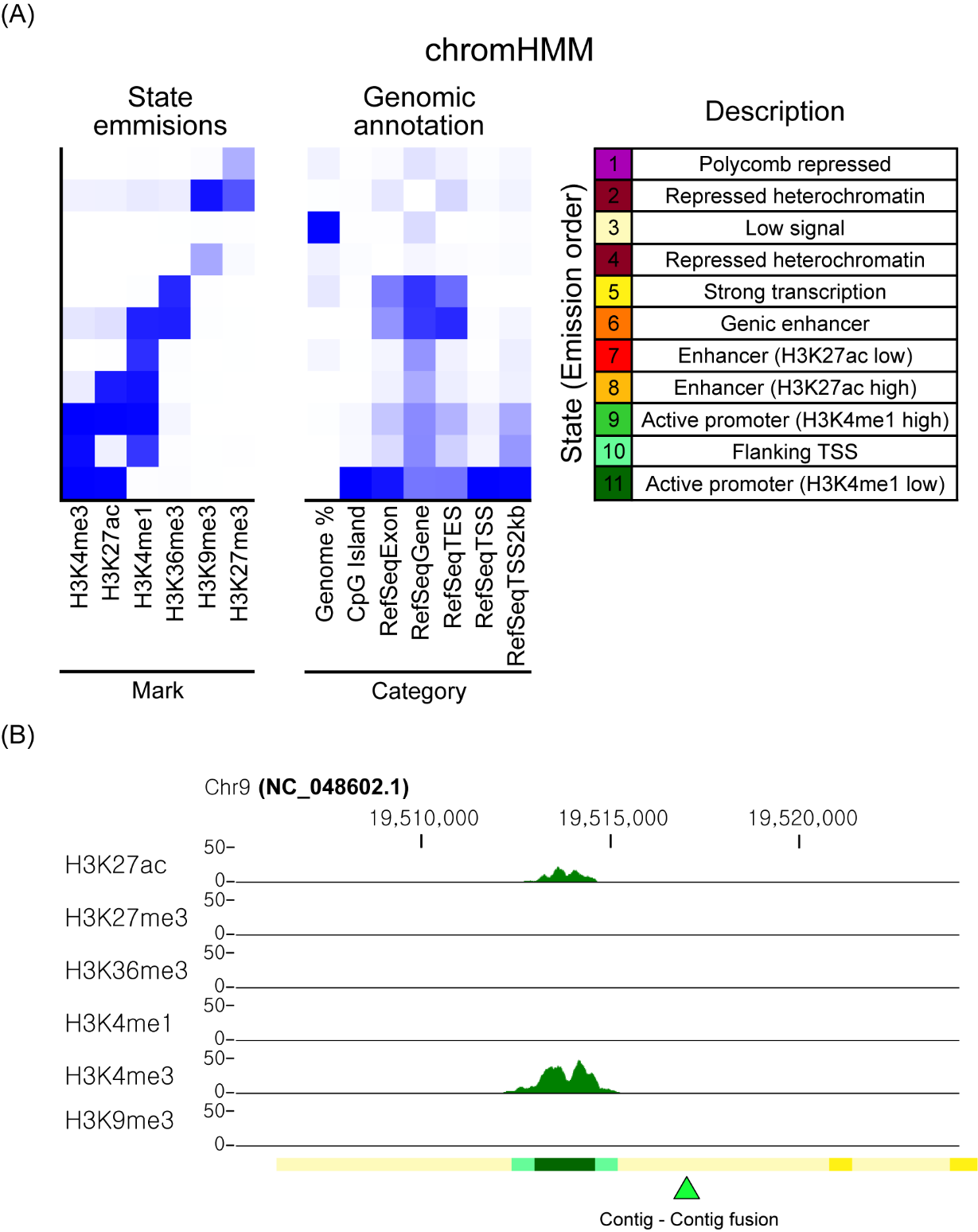


**Supplementary Figure 9**. (A) Chromatin state elucidated using chromHMM. Each row represents one chromatin state. From left to right: Histone mark and likelihood utilized to define the states (State emission). Chromatin state enrichment in genomic features (genomic annotation). Description of 11 states (Description). (B) GB tracks of ChIP-seq signal and chromHMM chromatin states around Construct integration site [2]. Y-axis represents normalized read counts.

**
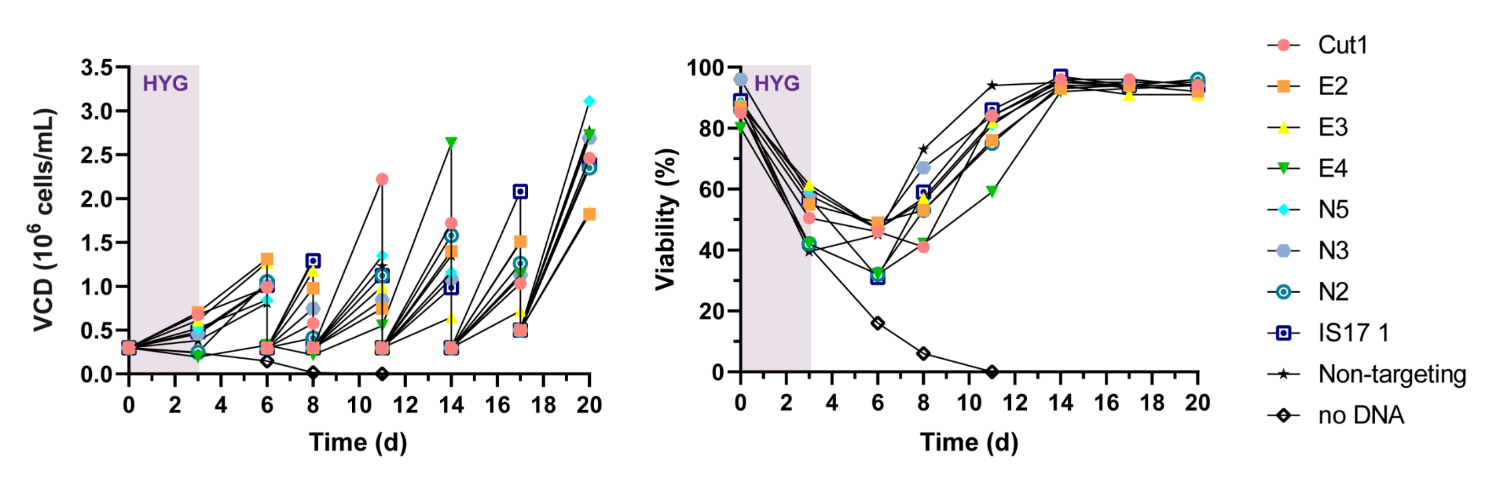
**

**Supplementary Figure 10**. Hygromycin selection profiles of indel mutation cell lines. Profiles of VCD and viability of sgRNA-targeting cells, non-targeting cells, and no-DNA controls during hygromycin selection. Hygromycin (200 μg/mL) selection was performed for the first 3 d, after which it was replaced with fresh media.


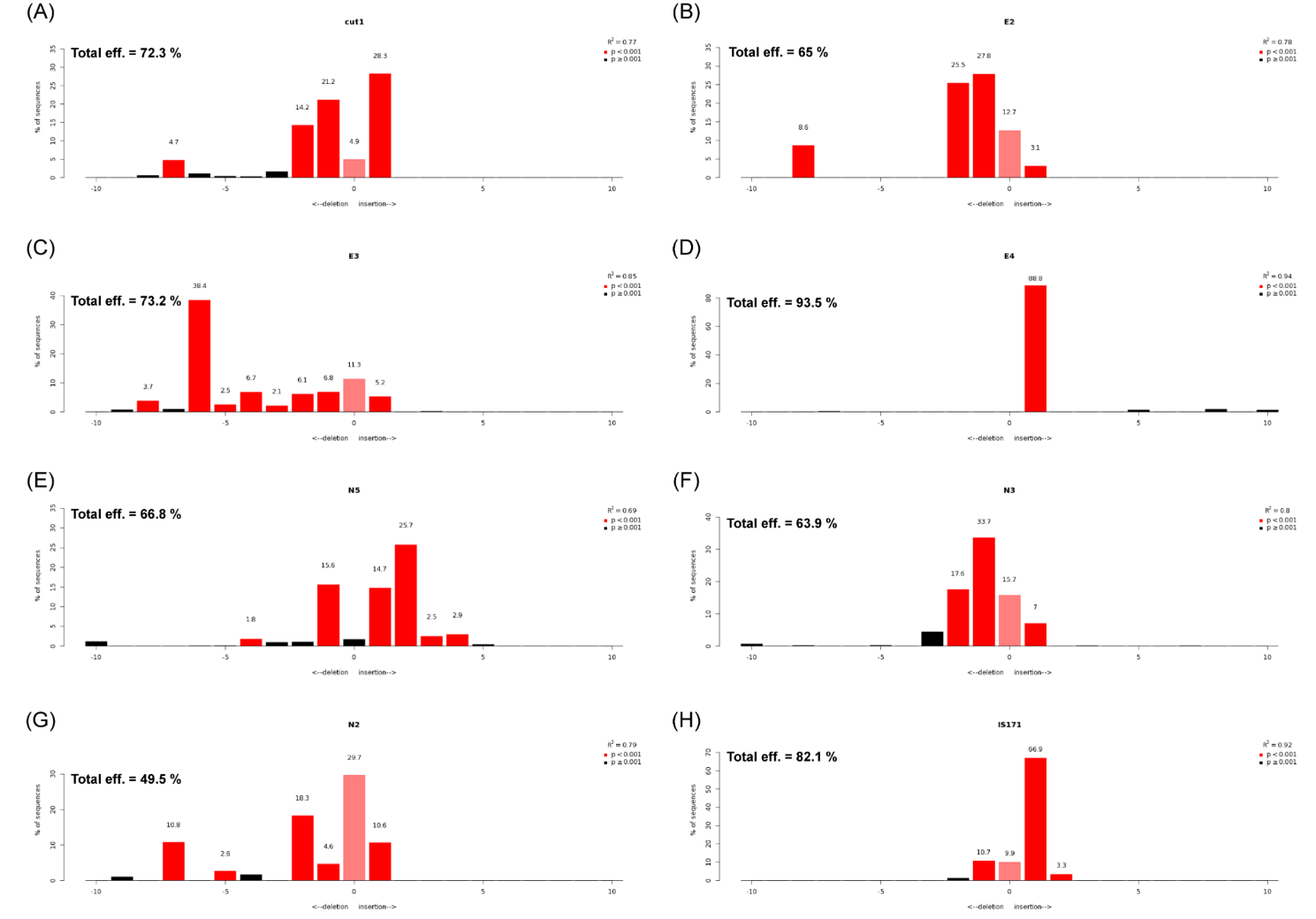


**Supplementary Figure 11.** Assessment of the indel frequency in indel mutation cell pools using TIDE analysis. Indel frequency of (A) Cut1, (B) E2, (C) E3, (D) E4, (E) N5, (F) N3, (G) N2, and (H) IS171 indel mutation cell pools were determined using TIDE analysis [3]. Target sites of all sgRNAs were compared to sgRNA non-targeting cells.


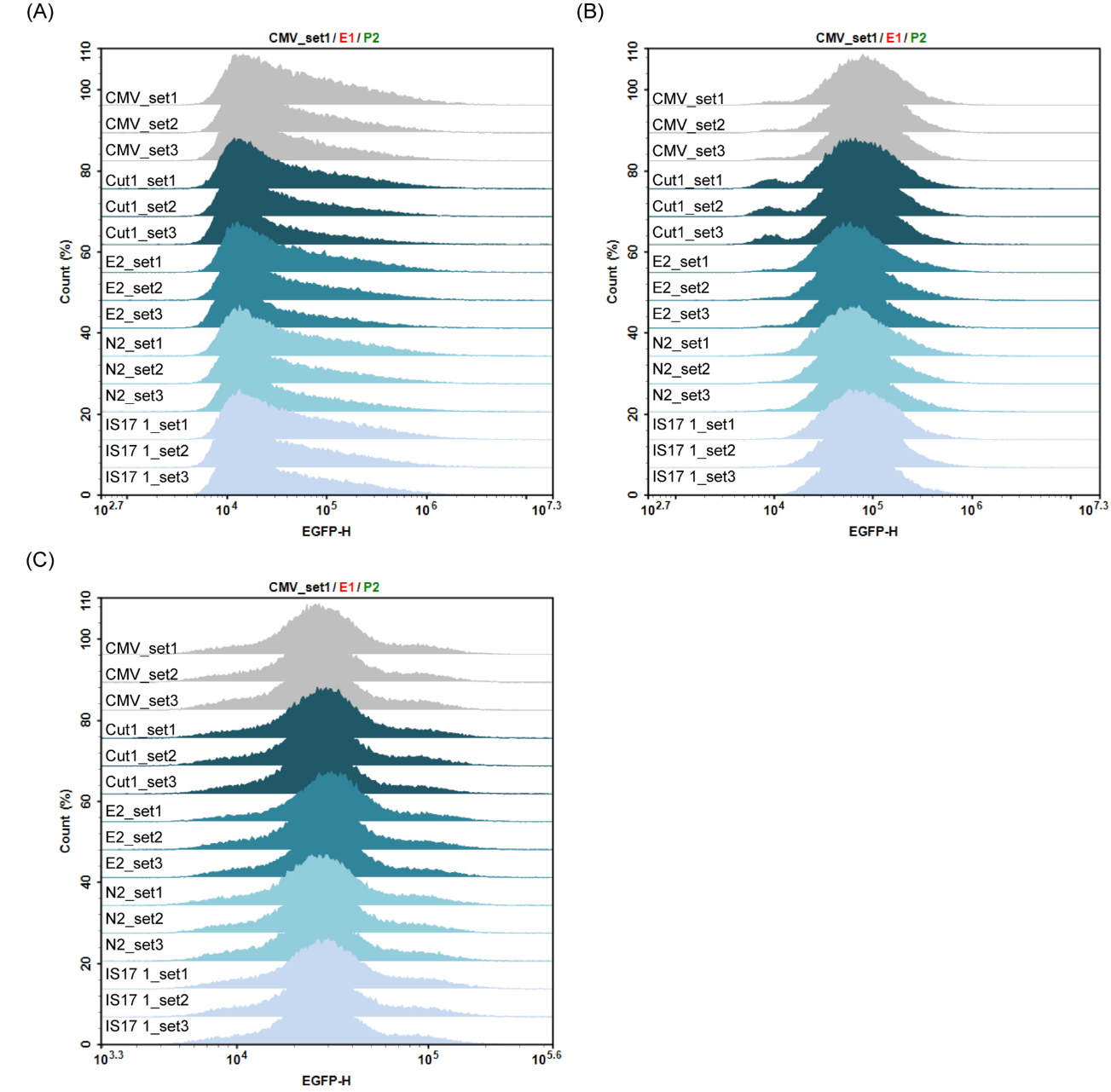


**Supplementary Figure 12.** Comparison of EGFP expression levels to confirm the effect of the IS14 sequence on transient and stable EGFP expression. Flow cytometry analysis of (A) EGFP transient expression, (B) EGFP stable expression generated through random integration, and (C) EGFP stable expression generated through promoter knock-in.


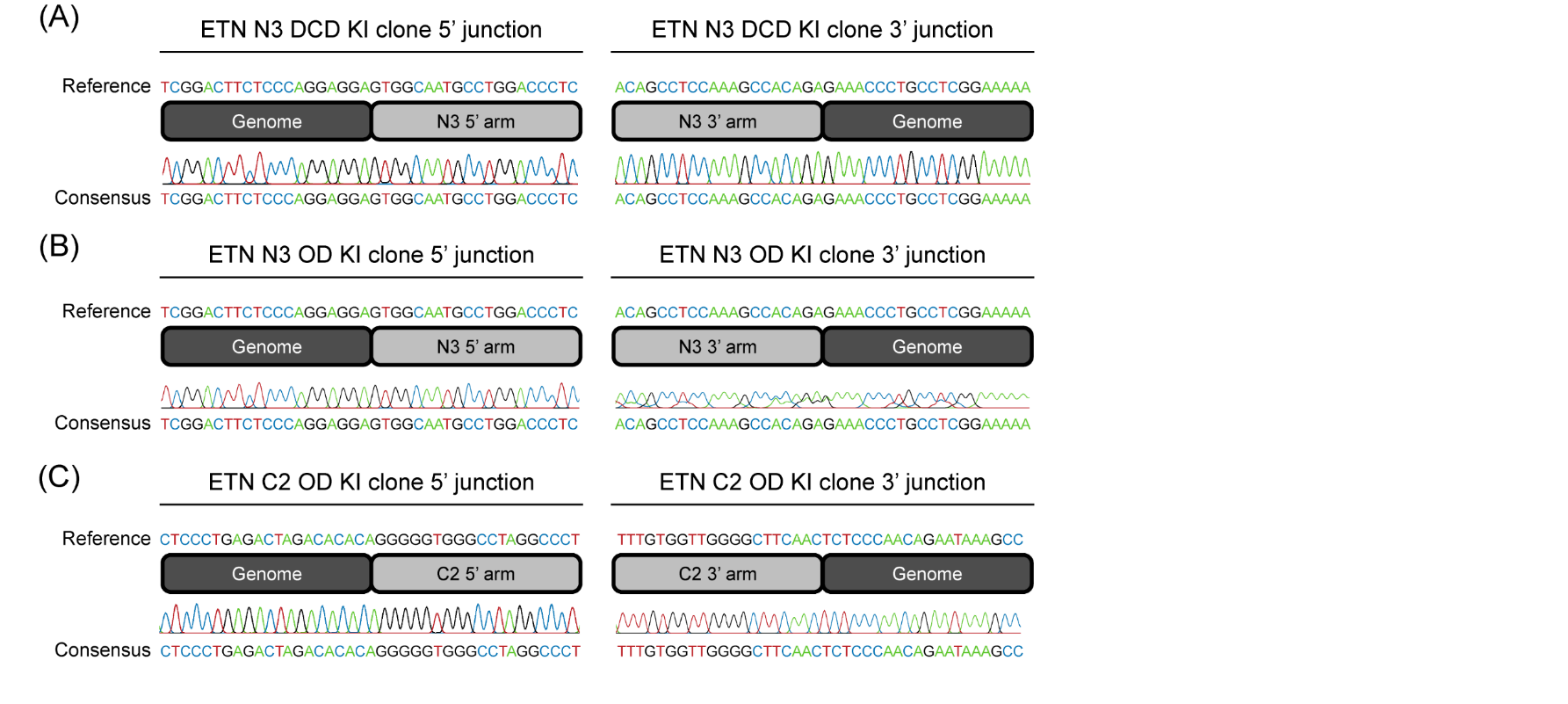


**Supplementary Figure 13**. Validation of ETN KI clone using Sanger sequencing. 5’/3’-junction PCR-positive amplicons of the (A) ETN N3 DCD KI, (B) ETN N3 OD KI, and (C) ETN C2 OD KI clones were verified using Sanger sequencing. Chromatogram sequence of the junction PCR amplicon was matched with the reference sequence to compare genome-donor boundaries.


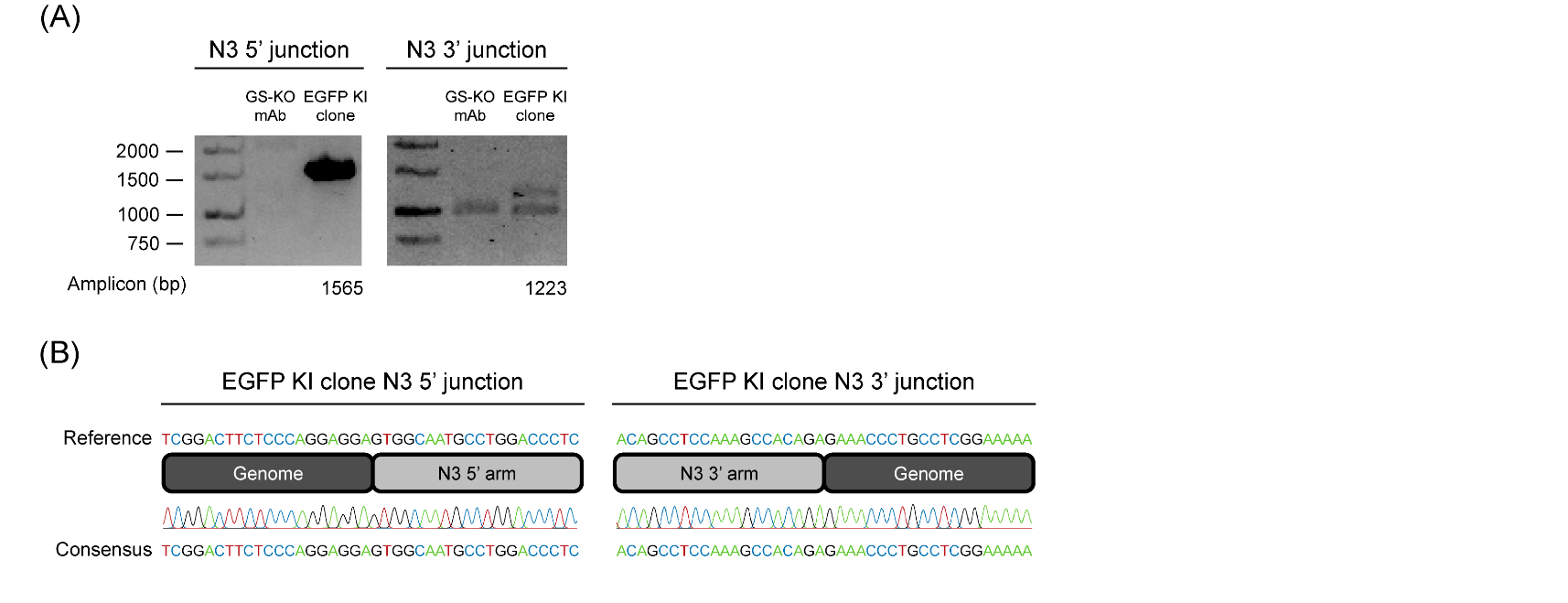


**Supplementary Figure 14.** Validation of the EGFP KI clone using 5’/3’-junction PCR and verification using Sanger sequencing. (A) 5’/3’-junction PCR analysis of EGFP N3 KI clone. (B) 5’/3’-junction PCR-positive amplicons of the EGFP N3 KI clone were verified using Sanger sequencing. Chromatogram sequence of the junction PCR amplicon was matched with the reference sequence to compare genome-donor boundaries.


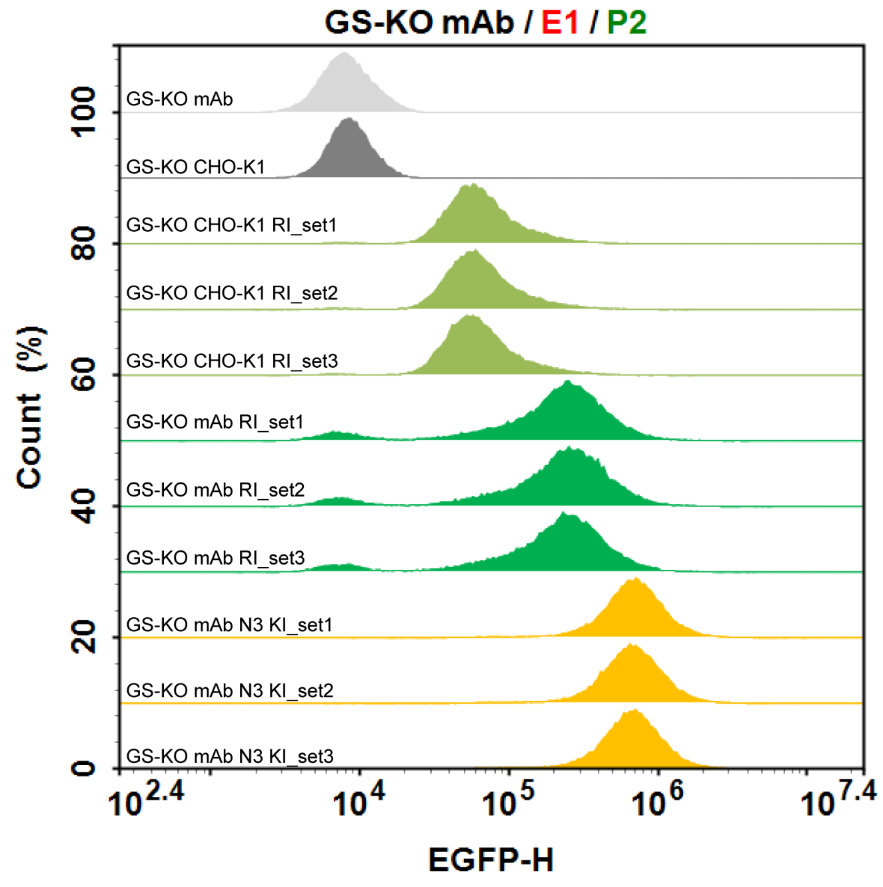


**Supplementary Figure 15**. Comparison of EGFP expression levels to evaluate expression capacity of the IS14 site in GS-KO mAb cells. Flow cytometry analysis of the control (GS-KO CHO-K1 and GS-KO mAb), GS-KO CHO-K1 EGFP RI pool, GS-KO mAb EGFP RI pool, and GS-KO mAb EGFP N3 KI clone.


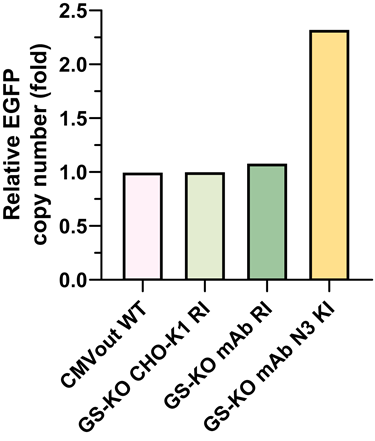


**Supplementary Figure 16.** Genotyping qPCR of EGFP gene copy number in GS-KO CHO-K1 EGFP RI pool, GS-KO mAb EGFP RI pool, and GS-KO mAb EGFP N3 KI clone. It was normalized to a reference cell line, CMVout WT, which contained a single-copy EGFP in the genome [4].

**Supplementary Figure 17.** Functional Gene Ontology (GO) analysis of copy number-gained genes in GS-KO mAb cells [5]. Based on the copy number analysis, the genes in GS-KO mAb cells were classified based on copy number gain and loss. Among the amplified genes in GS-KO mAb cells, nucleosome assembly related genes were functionally enriched.

**Supplementary Table 1. Plasmids used in this study**

| **Plasmid name** | **Description** | **Reference** |
| --- | --- | --- |
| Rituximab GS  random integration  donor | Plasmid donor for random integration of encoding heavy chain and light chains constituting mAb (Rituximab), glutamine synthetase, and zeocin resistance gene | [6] |
| Cas9-2A-mCherry | Cas9 2A peptide-linked mCherry without  specific targeting sgRNA expression control  (Addgene #64324) | [7] |
| Cas9-2A-HygR | Cas9 2A peptide-linked hygromycin resistance gene  without specific targeting sgRNA expression control  (Addgene #127763) | [8] |
| Non-targeting sgRNA  / Cas9-2A-HygR | Cas9 2A peptide-linked hygromycin resistance gene and Non-targeting sgRNA1 sgRNA of CHO-K1 wild type genome | This study |
| Cut1 sgRNA  / Cas9-2A-HygR | Cas9 2A peptide-linked hygromycin resistance gene and Cut1 sgRNA targeting IS14 site | This study |
| E2 sgRNA  / Cas9-2A-HygR | Cas9 2A peptide-linked hygromycin resistance gene and E2 sgRNA targeting IS14 site | This study |
| E3 sgRNA  / Cas9-2A-HygR | Cas9 2A peptide-linked hygromycin resistance gene and E3 sgRNA targeting IS14 site | This study |
| E4 sgRNA  / Cas9-2A-HygR | Cas9 2A peptide-linked hygromycin resistance gene and E4 sgRNA targeting IS14 site | This study |
| N5 sgRNA  / Cas9-2A-HygR | Cas9 2A peptide-linked hygromycin resistance gene and N5 sgRNA targeting IS14 site | This study |
| N3 sgRNA  / Cas9-2A-HygR | Cas9 2A peptide-linked hygromycin resistance gene and N3 sgRNA targeting IS14 site | This study |
| N2 sgRNA  / Cas9-2A-HygR | Cas9 2A peptide-linked hygromycin resistance gene and N2 sgRNA targeting IS14 site | This study |
| IS17 1 sgRNA  / Cas9-2A-HygR | Cas9 2A peptide-linked hygromycin resistance gene and IS17 1 sgRNA targeting IS14 site | This study |
| EGFP random integration  donor | Plasmid donor for random integration of  CMV-EGFP-2A-puromycin resistance gene | This study |
| Cut1 EGFP  random integration  donor | Plasmid donor for random integration of  Cut1-CMV-EGFP-2A  puromycin resistance gene | This study |
| E2 EGFP  random integration  donor | Plasmid donor for random integration of  E2-CMV-EGFP-2A-puromycin resistance gene | This study |
| N2 EGFP  random integration  donor | Plasmid donor for random integration of  N2-CMV-EGFP-2A-puromycin resistance gene | This study |
| IS17 1 EGFP  random integration  donor | Plasmid donor for random integration of  IS17 1-CMV-EGFP-2A-puromycin resistance gene | This study |
| sgRNA1 sgRNA  / Cas9-2A-mCherry | Cas9 2A peptide-linked mCherry and sgRNA1  sgRNA targeting monitoring cell line | [9] |
| CMV KI donor | Plasmid donor for targeted integration of the  5’ homology arm-CMV-3’ homology arm  into the monitoring cell line | This study |
| Cut1 CMV KI donor | Plasmid donor for targeted integration of the  5’ homology arm-Cut1-CMV-3’ homology arm  into the monitoring cell line | This study |
| E2 CMV KI donor | Plasmid donor for targeted integration of the  5’ homology arm-E2-CMV-3’ homology arm  into the monitoring cell line | This study |
| N2 CMV KI donor | Plasmid donor for targeted integration of the  5’ homology arm-N2-CMV-3’ homology arm  into the monitoring cell line | This study |
| IS17 1 CMV KI donor | Plasmid donor for targeted integration of the  5’ homology arm-IS17 1-CMV-3’ homology arm  into the monitoring cell line | This study |
| N3 sgRNA  / Cas9-2A-mCherry | Cas9 2A peptide-linked mCherry and N3 sgRNA  targeting IS14 site | This study |
| C2 sgRNA  / Cas9-2A- mCherry | Cas9 2A peptide-linked mCherry and C2 sgRNA  targeting IS14 site | This study |
| ETN N3 KI DCD donor | Double cut donor for targeted integration of the  5’ homology arm-ETN-zeocin resistance gene  -3’ homology arm into the IS14 N3 site | This study |
| ETN N3 KI OD donor | Plasmid donor for targeted integration of the  5’ homology arm-ETN-zeocin resistance gene  -3’ homology arm into the IS14 N3 site | This study |
| ETN C2 KI OD donor | Plasmid donor for targeted integration of the  5’ homology arm-ETN-zeocin resistance gene  -3’ homology arm into the IS14 C2 site | This study |
| EGFP N3 KI donor | Plasmid donor for targeted integration of the  5’ homology arm-EGFP-2A-puromycin resistance gene  -3’ homology arm-TagBFP into the IS14 N3 site | This study |

**Supplementary Table 2. Primer sequences used in this study**

| **Constructing sgRNA/Cas9 expression vector and donor plasmid** | | |
| --- | --- | --- |
| **Primer name** | **Component** | **Sequence (5'-3')** |
| cutIS14_1_fwd | Cut1 sgRNA/Cas9 vector | CACCGGCATCAGAAAGTACGGCCAT |
| cutIS14_1_rev |  | AAACATGGCCGTACTTTCTGATGCC |
| sgIS14E_2_fwd | E2 sgRNA/Cas9 vector | CACCGACTCTAGAGCCTCTTTCCCT |
| sgIS14E_2_rev |  | AAACAGGGAAAGAGGCTCTAGAGTC |
| sgIS14E_3_fwd | E3 sgRNA/Cas9 vector | CACCGCTCAGACGGAAAATTGGTAT |
| sgIS14E_3_rev |  | AAACATACCAATTTTCCGTCTGAGC |
| sgIS14E_4_fwd | E4 sgRNA/Cas9 vector | CACCGAGTATAGAATGATGTTAGCA |
| sgIS14E_4_rev |  | AAACTGCTAACATCATTCTATACTC |
| sgIS14N_5_fwd | N5 sgRNA/Cas9 vector | CACCGCCGAGGCCTTTGTACCGGAG |
| sgIS14N_5_rev |  | AAACCTCCGGTACAAAGGCCTCGGC |
| sgIS14N_3_fwd | N3 sgRNA/Cas9 vector | CACCGAGAGATTGTTTAGAAGCCCC |
| sgIS14N_3_rev |  | AAACGGGGCTTCTAAACAATCTCTC |
| sgIS14N_2_fwd | N2 sgRNA/Cas9 vector | CACCGATTCATTCACACCACTGACC |
| sgIS14N_2_rev |  | AAACGGTCAGTGGTGTGAATGAATC |
| sgIS17_1_fwd | IS17 1  sgRNA/Cas9 vector | CACCGTATGCTTCTTCCTCATTTGA |
| sgIS17_1_rev |  | AAACTCAAATGAGGAAGAAGCATAC |
| sgIS14C_2_fwd | C2  sgRNA/Cas9 vector | CACCGCCACAGCAGTGGAAGACTCC |
| sgIS14C_2_rev |  | AAACGGAGTCTTCCACTGCTGTGGC |
| sgRNA1_fwd | sgRNA1  sgRNA/Cas9 vector | CACCGCGAAGGCTACGTCCATATAT |
| sgRNA1_rev |  | AAACATATATGGACGTAGCCTTCGC |
| pcDNA3.1_BB_DB_rev | EGFP  random integration  donor | ACGATGGCCUCAGTCGGGAAACCTGTCGTG |
| CMV_DB_fwd |  | AGGCCATCGUGTTGACATTGATTATTGACT |
| IS14CMV_LC_rev |  | ATGACGTCUGAGCTCTGCTTATATAGACC |
| EGFP_LC_fwd |  | AGACGTCAUCGCCACCATGGTGAGCAAGG |
| BGHpA_O5_rev |  | ACGCAAGUCCATAGAGCCCACCGCATC |
| pcDNA3.1_BB_O5_fwd |  | ACTTGCGUACTTTTCGGGGAAATGTGCG |
| pcDNA3.1_BB_LF_rev | Cut1 EGFP  random integration  donor | ATCCACGTUCAGTCGGGAAACCTGTCGTG |
| Cut1_LF_fwd |  | AACGTGGAUGTAACCCCTGTCCCCAAAGG |
| Cut1_DB_rev |  | ACGATGGCCUCTCTCCAGCCCCCGAAATTA |
| CMV_DB_fwd |  | AGGCCATCGUGTTGACATTGATTATTGACT |
| IS14CMV_LC_rev |  | ATGACGTCUGAGCTCTGCTTATATAGACC |
| EGFP_LC_fwd |  | AGACGTCAUCGCCACCATGGTGAGCAAGG |
| BGHpA_O5_rev |  | ACGCAAGUCCATAGAGCCCACCGCATC |
| pcDNA3.1_BB_O5_fwd |  | ACTTGCGUACTTTTCGGGGAAATGTGCG |
| pcDNA3.1_BB_LF_rev | E2 EGFP  random integration  donor | ATCCACGTUCAGTCGGGAAACCTGTCGTG |
| E2_LF_fwd |  | AACGTGGAUTGGAGAGATGGCTCAGAAGT |
| E2_DB_rev |  | ACGATGGCCUTGCAGCCAGGAGGAAGCTGC |
| CMV_DB_fwd |  | AGGCCATCGUGTTGACATTGATTATTGACT |
| IS14CMV_LC_rev |  | ATGACGTCUGAGCTCTGCTTATATAGACC |
| EGFP_LC_fwd |  | AGACGTCAUCGCCACCATGGTGAGCAAGG |
| BGHpA_O5_rev |  | ACGCAAGUCCATAGAGCCCACCGCATC |
| pcDNA3.1_BB_O5_fwd |  | ACTTGCGUACTTTTCGGGGAAATGTGCG |
| pcDNA3.1_BB_LF_rev | N2 EGFP  random integration  donor | ATCCACGTUCAGTCGGGAAACCTGTCGTG |
| N2_LF_fwd |  | AACGTGGAUAGAAGTGGGACATGAAATTT |
| N2_DB_rev |  | ACGATGGCCUCCGAGGCAGGGTTTCTCTGT |
| CMV_DB_fwd |  | AGGCCATCGUGTTGACATTGATTATTGACT |
| IS14CMV_LC_rev |  | ATGACGTCUGAGCTCTGCTTATATAGACC |
| EGFP_LC_fwd |  | AGACGTCAUCGCCACCATGGTGAGCAAGG |
| BGHpA_O5_rev |  | ACGCAAGUCCATAGAGCCCACCGCATC |
| pcDNA3.1_BB_O5_fwd |  | ACTTGCGUACTTTTCGGGGAAATGTGCG |
| pcDNA3.1_BB_LF_rev | IS17 1 EGFP  random integration  donor | ATCCACGTUCAGTCGGGAAACCTGTCGTG |
| IS17 1_LF_fwd |  | AACGTGGAUGGATAGGTAGGGTTTTAGTT |
| IS17 1_DB_rev |  | ACGATGGCCUTTCACCTGACCTAGTAAGAG |
| CMV_DB_fwd |  | AGGCCATCGUGTTGACATTGATTATTGACT |
| IS14CMV_LC_rev |  | ATGACGTCUGAGCTCTGCTTATATAGACC |
| EGFP_LC_fwd |  | AGACGTCAUCGCCACCATGGTGAGCAAGG |
| BGHpA_O5_rev |  | ACGCAAGUCCATAGAGCCCACCGCATC |
| pcDNA3.1_BB_O5_fwd |  | ACTTGCGUACTTTTCGGGGAAATGTGCG |
| pcDNA3.1_BB _LA_rev | CMV KI donor | ATGACGTCUGTACAGCTCGTCCATGCCG |
| monitoring_5arm_LA_fwd |  | AGTCGGTGUAAGGCATGCACCACCACCAC |
| monitoring _5arm_DB_rev |  | ACGATGGCCUCTATGAACTAATGACCCCGT |
| CMV_DB_fwd |  | AGGCCATCGUGTTGACATTGATTATTGACT |
| IS14CMV_LC_rev |  | ATGACGTCUGAGCTCTGCTTATATAGACC |
| EGFP_LC_fwd |  | AGACGTCAUCGCCACCATGGTGAGCAAGG |
| monitoring_3arm_O5_rev |  | ACGCAAGUGCTTGAGCTCGAGATCTGAG |
| pcDNA3.1_BB_O5_fwd |  | ACTTGCGUACTTTTCGGGGAAATGTGCG |
| pcDNA3.1_BB _LA_rev | Cut1 CMV KI donor | ATGACGTCUGTACAGCTCGTCCATGCCG |
| monitoring_5arm_LA_fwd |  | AGTCGGTGUAAGGCATGCACCACCACCAC |
| monitoring _5arm_DB_rev |  | ACGATGGCCUCTATGAACTAATGACCCCGT |
| monitoring_5arm_LF_rev |  | ATCCACGTUCTATGAACTAATGACCCCGT |
| Cut1_LF_fwd |  | AACGTGGAUGTAACCCCTGTCCCCAAAGG |
| Cut1_DB_rev |  | ACGATGGCCUCTCTCCAGCCCCCGAAATTA |
| CMV_DB_fwd |  | AGGCCATCGUGTTGACATTGATTATTGACT |
| IS14CMV_LC_rev |  | ATGACGTCUGAGCTCTGCTTATATAGACC |
| EGFP_LC_fwd |  | AGACGTCAUCGCCACCATGGTGAGCAAGG |
| monitoring_3arm_O5_rev |  | ACGCAAGUGCTTGAGCTCGAGATCTGAG |
| pcDNA3.1_BB_O5_fwd |  | ACTTGCGUACTTTTCGGGGAAATGTGCG |
| pcDNA3.1_BB _LA_rev | E2 CMV KI donor | ATGACGTCUGTACAGCTCGTCCATGCCG |
| monitoring_5arm_LA_fwd |  | AGTCGGTGUAAGGCATGCACCACCACCAC |
| monitoring _5arm_DB_rev |  | ACGATGGCCUCTATGAACTAATGACCCCGT |
| monitoring_5arm_LF_rev |  | ATCCACGTUCTATGAACTAATGACCCCGT |
| E2_LF_fwd |  | AACGTGGAUTGGAGAGATGGCTCAGAAGT |
| E2_DB_rev |  | ACGATGGCCUTGCAGCCAGGAGGAAGCTGC |
| CMV_DB_fwd |  | AGGCCATCGUGTTGACATTGATTATTGACT |
| IS14CMV_LC_rev |  | ATGACGTCUGAGCTCTGCTTATATAGACC |
| EGFP_LC_fwd |  | AGACGTCAUCGCCACCATGGTGAGCAAGG |
| monitoring_3arm_O5_rev |  | ACGCAAGUGCTTGAGCTCGAGATCTGAG |
| pcDNA3.1_BB_O5_fwd |  | ACTTGCGUACTTTTCGGGGAAATGTGCG |
| pcDNA3.1_BB _LA_rev | N2 CMV KI donor | ATGACGTCUGTACAGCTCGTCCATGCCG |
| monitoring_5arm_LA_fwd |  | AGTCGGTGUAAGGCATGCACCACCACCAC |
| monitoring _5arm_DB_rev |  | ACGATGGCCUCTATGAACTAATGACCCCGT |
| monitoring_5arm_LF_rev |  | ATCCACGTUCTATGAACTAATGACCCCGT |
| N2_LF_fwd |  | AACGTGGAUAGAAGTGGGACATGAAATTT |
| N2_DB_rev |  | ACGATGGCCUCCGAGGCAGGGTTTCTCTGT |
| CMV_DB_fwd |  | AGGCCATCGUGTTGACATTGATTATTGACT |
| IS14CMV_LC_rev |  | ATGACGTCUGAGCTCTGCTTATATAGACC |
| EGFP_LC_fwd |  | AGACGTCAUCGCCACCATGGTGAGCAAGG |
| monitoring_3arm_O5_rev |  | ACGCAAGUGCTTGAGCTCGAGATCTGAG |
| pcDNA3.1_BB_O5_fwd |  | ACTTGCGUACTTTTCGGGGAAATGTGCG |
| pcDNA3.1_BB _LA_rev | IS17 1 CMV KI donor | ATGACGTCUGTACAGCTCGTCCATGCCG |
| monitoring_5arm_LA_fwd |  | AGTCGGTGUAAGGCATGCACCACCACCAC |
| monitoring _5arm_DB_rev |  | ACGATGGCCUCTATGAACTAATGACCCCGT |
| monitoring_5arm_LF_rev |  | ATCCACGTUCTATGAACTAATGACCCCGT |
| IS17 1_LF_fwd |  | AACGTGGAUGGATAGGTAGGGTTTTAGTT |
| IS17 1_DB_rev |  | ACGATGGCCUTTCACCTGACCTAGTAAGAG |
| CMV_DB_fwd |  | AGGCCATCGUGTTGACATTGATTATTGACT |
| IS14CMV_LC_rev |  | ATGACGTCUGAGCTCTGCTTATATAGACC |
| EGFP_LC_fwd |  | AGACGTCAUCGCCACCATGGTGAGCAAGG |
| monitoring_3arm_O5_rev |  | ACGCAAGUGCTTGAGCTCGAGATCTGAG |
| pcDNA3.1_BB_O5_fwd |  | ACTTGCGUACTTTTCGGGGAAATGTGCG |
| pcDNA3.1_BB _LA_rev | ETN N3 KI DCD donor | ATGACGTCUGTACAGCTCGTCCATGCCG |
| N3_KI_5arm_sgIS14N3  /PAM_LA_fwd |  | AGTCGGTGUAGAGATTGTTTAGAAGCCCCTGGGTGGCAATGCCTGGACCCTC |
| N3_KI_5arm_DB_rev |  | ACGATGGCCUCCCTGGATTTCTCACACAGT |
| CMV_DB_fwd |  | AGGCCATCGUGTTGACATTGATTATTGACT |
| SV40pA_LF_rev |  | AACGTGGAUGTGTGTCAGTTAGGGTGTGG |
| N3_KI_3arm _LF_fwd |  | ATCCACGTUGCTTCTAAACAATCTCTTGC |
| N3_KI_3arm_sgIS14N3  /PAM_O5_rev |  | ACGCAAGUAGAGATTGTTTAGAAGCCCCTGGTCTGTGGCTTTGGAGGCTGT |
| pcDNA3.1_BB_O5_fwd |  | ACTTGCGUACTTTTCGGGGAAATGTGCG |
| pcDNA3.1_BB _LA_rev | ETN N3 KI OD donor | ATGACGTCUGTACAGCTCGTCCATGCCG |
| N3_KI_5arm_LA_fwd |  | AGTCGGTGUGTGGCAATGCCTGGACCCTC |
| N3_KI_5arm_DB_rev |  | ACGATGGCCUCCCTGGATTTCTCACACAGT |
| CMV_DB_fwd |  | AGGCCATCGUGTTGACATTGATTATTGACT |
| SV40pA_LF_rev |  | AACGTGGAUGTGTGTCAGTTAGGGTGTGG |
| N3_KI_3arm _LF_fwd |  | ATCCACGTUGCTTCTAAACAATCTCTTGC |
| N3_3arm_O5_rev |  | ACGCAAGUTCTGTGGCTTTGGAGGCTGT |
| pcDNA3.1_BB_O5_fwd |  | ACTTGCGUACTTTTCGGGGAAATGTGCG |
| pcDNA3.1_BB _LA_rev | ETN C2 KI OD donor | ATGACGTCUGTACAGCTCGTCCATGCCG |
| C2_KI_5arm _LA_fwd |  | AGTCGGTGUAGGGGGTGGGCCTAGGCCCT |
| C2_KI_5arm _DB_rev |  | ACGATGGCCUTCCAGGTTTTGCTTCTTGTG |
| CMV_DB_fwd |  | AGGCCATCGUGTTGACATTGATTATTGACT |
| SV40pA_LF_rev |  | AACGTGGAUGTGTGTCAGTTAGGGTGTGG |
| C2_KI_3arm _LF_fwd |  | ATCCACGTUGTCTTCCACTGCTGTGGCCT |
| C2_KI_3arm _O5_rev |  | ACGCAAGUGTTGAAGCCCCAACCACAAA |
| pcDNA3.1_BB_O5_fwd |  | ACTTGCGUACTTTTCGGGGAAATGTGCG |
| pcDNA3.1_BB _DB_rev | TagBFP pcDNA3.1 | ACGATGGCCUCAGTCGGGAAACCTGTCGTG |
| CMV_DB_fwd |  | AGGCCATCGUGTTGACATTGATTATTGACT |
| IS14CMV_LC_rev |  | ATGACGTCUGAGCTCTGCTTATATAGACC |
| Kozak_TagBFP_LC_fwd |  | AGACGTCAUGCCACCATGAGCGAGCTGATTAAGGA |
| SV40pA_O5_rev |  | ACGCAAGUTAAGATACATTGATGAGTTTG |
| pcDNA3.1_BB_O5_fwd |  | ACTTGCGUACTTTTCGGGGAAATGTGCG |
| pcDNA3.1_BB _LA_rev | EGFP N3 KI donor with TagBFP pcDNA3.1 backbone | ATGACGTCUGTACAGCTCGTCCATGCCG |
| N3_KI_5arm_LA_fwd |  | AGTCGGTGUGTGGCAATGCCTGGACCCTC |
| N3_KI_5arm_DB_rev |  | ACGATGGCCUCCCTGGATTTCTCACACAGT |
| CMV_DB_fwd |  | AGGCCATCGUGTTGACATTGATTATTGACT |
| BGHpA_LF_rev |  | AACGTGGAUCCATAGAGCCCACCGCATCC |
| N3_KI_3arm _LF_fwd |  | ATCCACGTUGCTTCTAAACAATCTCTTGC |
| N3_KI_3arm _O5_rev |  | ACGCAAGUTCTGTGGCTTTGGAGGCTGT |
| SV40E _O5_fwd |  | ACTTGCGUCTGTGGAATGTGTGTCAGTT |
| SV40E_LC_rev |  | ATGACGTCUGTGCTGATCAGATCCGAAAATGG |
| Kozak_TagBFP_LC_fwd |  | AGACGTCAUGCCACCATGAGCGAGCTGATTAAGGA |
| **IS14 site validation in GS-KO mAb cell genome** | | |
| **Primer name** | **Component** | **Sequence (5'-3')** |
| IS14_5Junc_fwd2 | Construct 1 validation  in IS14 site  (1 fwd - 1 rev) | CTTCCGTACTCAAAAGCGTGG |
| sgIS14N_TIDE_fwd |  | TCTGAGGCGTGACCTCTAGCA |
| IS14CB_5Junc_seq_fwd |  | CTTCGGACTTCTCCCAGGAGGA |
| IS14CB_5Junc_seq_rev |  | CTTTCATGGGGTCTGGATCAGT |
| IS14_5Junc_fwd1 |  | TGAAAACTTTGTTAGTCCAGCTTCT |
| IS17_5Junc_rev1 |  | AGCACTGATTAGCAGGAAGC |
| mAb_LC_qPCR_rev |  | TCCTGCTCTGTGACACTCT |
| mAb_LC_qPCR_fwd |  | GTTGTGTGCCTGCTGAATAAC |
| GS_Junc_fwd |  | TTAGGGTTAGGCGTTTTGCG |
| GS_Junc_rev |  | ATACAAGCAGGCGCGGTAG |
| IS14_GS_HC_fwd |  | AGCTCATTTTTTAACCAATA |
| IS17_5Junc_rev2 |  | GTACACCTGTGGTTCTCGGG |
| mAb_HC_fwd | Construct 1 validation  in IS14 site  (2 fwd - 2 rev) | GGGTTGGAGCCTCATCTTGCTCT |
| mAb_HC_qPCR_rev |  | CATCACGGAGCATGAGAAGA |
| mAb_HC_qPCR_fwd |  | CAGCCGGAGAACAACTACAA |
| ZeoR_seq_rev |  | TGATGAACAGGGTCACGTCG |
| SV40p LF fwd | Construct 1 validation  in IS14 site  (3 fwd - 3 rev) | AACGTGGAUGTGTGTCAGTTAGGGTGTGG |
| Ori_BB_LE_fwd |  | ATAGGCTTUTAATCTCATGACCAAAATCC |
| GS_mAb_rev |  | TCAACATTTCCGTGTCGCCCT |
| IS14_5Junc_fwd1 | Construct 2 validation  in IS14 site  (4 fwd - 4 rev) | TGAAAACTTTGTTAGTCCAGCTTCT |
| IS17_5Junc_rev1 |  | AGCACTGATTAGCAGGAAGC |
| mAb_LC_qPCR_rev |  | TCCTGCTCTGTGACACTCT |
| mAb_LC_qPCR_fwd |  | GTTGTGTGCCTGCTGAATAAC |
| ZeoR_seq_rev |  | TGATGAACAGGGTCACGTCG |
| mAb_LC_qPCR_fwd | Construct 2 validation  in IS14 site  (5 fwd - 5 rev) | GTTGTGTGCCTGCTGAATAAC |
| ZeoR_seq_rev |  | TGATGAACAGGGTCACGTCG |
| GS_Junc_fwd |  | TTAGGGTTAGGCGTTTTGCG |
| ZeoR_seq_fwd | Construct 2 validation  in IS14 site  (6 fwd - 6 rev) | GTCCACGAACTTCCGGGACG |
| mAb_LC_junc_rev |  | GCCCTCCAATCGGGTAACT |
| **Quantitative real-time PCR** | | |
| **Primer name** | **Component** | **Sequence (5'-3')** |
| IS14_qPCR_fwd | Amplicon for IS14 site  (NW_003614743.1) | CGATTCTCTTTGCTGTGGTGC |
| IS14_qPCR_rev |  | CCACGCTTTTGAGTACGGAAG |
| IS17_qPCR_fwd | Amplicon for IS17 site  (NW_023276964.1) | CCAAACCACCTGCCACAACC |
| IS17_qPCR_rev |  | GTCCAGACACAGGAAGAAGG |
| EGFP_fwd | Amplicon for EGFP | GAACCGCATCGAGCTGAA |
| EGFP_rev |  | TGCTTGTCGGCCATGATATAG |
| **TIDE analysis** | | |
| **Primer name** | **component** | **Sequence (5'-3')** |
| IS14_5Junc_fwd3 | Amplicon for  Cut1 and E2 sgRNA target sequence | AGGTTGACTATGTATAATGG |
| IS14_TIDE_rev1 |  | TAGACCGTTGTCCCCGCC |
| IS14_5Junc_fwd2 | Amplicon for  E3 and E4 sgRNA target sequence | CTTCCGTACTCAAAAGCGTGG |
| IS14_5Junc_rev |  | CGCCTCAGACACAGCAAACA |
| sgIS14N_TIDE_fwd | Amplicon for  N5, N3, and N2 sgRNA  target sequence | TCTGAGGCGTGACCTCTAGCA |
| sgIS14N_TIDE_rev |  | CCAACAGACAACAGGAGACAGTAT |
| IS14_5Junc_fwd1 | Amplicon for  IS17 1 sgRNA  target sequence | TGAAAACTTTGTTAGTCCAGCTTCT |
| IS14_TIDE_rev2 |  | CGTCAATAGGGGGCGTACTT |
| **5’/3’-junction PCR** | | |
| **Primer name** | **Component** | **Sequence (5'-3')** |
| sgIS14N_TIDE_fwd | 5’ junction for  ETN N3 DCD KI | TCTGAGGCGTGACCTCTAGCA |
| CMV_qPCR_rev |  | CTATTGGCGTTACTATGGGAACATAC |
| ZeoR_Seq_rev | 3’ junction for  ETN N3 DCD KI | TGATGAACAGGGTCACGTCG |
| sgIS14N_TIDE_rev |  | CCAACAGACAACAGGAGACAGTAT |
| sgIS14N_TIDE_fwd | 5’ junction for  ETN N3 OD KI | TCTGAGGCGTGACCTCTAGCA |
| CMV_qPCR_rev |  | CTATTGGCGTTACTATGGGAACATAC |
| ZeoR_Seq_rev | 3’ junction for  ETN N3 OD KI | TGATGAACAGGGTCACGTCG |
| sgIS14N_TIDE_rev |  | CCAACAGACAACAGGAGACAGTAT |
| IS14_5Junc_fwd1 | 5’ junction for  ETN C2 OD KI | TGAAAACTTTGTTAGTCCAGCTTCT |
| CMV_qPCR_rev |  | CTATTGGCGTTACTATGGGAACATAC |
| ZeoR_Seq_rev | 3’ junction for  ETN C2 OD KI | TGATGAACAGGGTCACGTCG |
| IS14_3Junc_rev |  | GCCCCAAGGCTTCAAGGAGCCGA |
| sgIS14N_TIDE_fwd | 5’ junction for  EGFP N3 KI | TCTGAGGCGTGACCTCTAGCA |
| EGFP_rev |  | GCGGACTTGAAGAAGTCGTG |
| PuroR_fwd | 3’ junction for  EGFP N3 KI | CACCAGGGCAAGGGTCTG |
| sgIS14N_TIDE_rev |  | CCAACAGACAACAGGAGACAGTAT |

**References**

[1] Talevich E, Shain AH, Botton T, Bastian BC (2016) CNVkit: Genome-Wide Copy Number Detection and Visualization from Targeted DNA Sequencing. PLoS Comput Biol 12:e1004873.

[2] Kent WJ, Sugnet CW, Furey TS, Roskin KM, Pringle TH, Zahler AM, Haussler D (2002) The human genome browser at UCSC. Genome Res 12: 996-1006.

[3] Brinkman EK, Chen T, Amendola M, Van Steensel B (2014) Easy quantitative assessment of genome editing by sequence trace decomposition. Nucleic Acids Res 42: e168-e168.

[4] Lee JS, Park JH, Ha TK, Samoudi M, Lewis NE, Palsson BO, Kildegaard HF, Lee GM (2018) Revealing key determinants of clonal variation in transgene expression in recombinant CHO cells using targeted genome editing. ACS synthetic biology 7: 2867-2878.

[5] Dennis G Jr, Sherman BT, Hosack DA, Yang J, Gao W, Lane HC, Lempicki RA (2003) DAVID: Database for Annotation, Visualization, and Integrated Discovery. Genome Biol 4:P3.

[6] Noh SM, Shin S, Lee GM (2018) Comprehensive characterization of glutamine synthetase-mediated selection for the establishment of recombinant CHO cells producing monoclonal antibodies. Scientific Reports 8: 5361.

[7] Chu VT, Weber T, Wefers B, Wurst W, Sander S, Rajewsky K, Kühn R (2015) Increasing the efficiency of homology-directed repair for CRISPR-Cas9-induced precise gene editing in mammalian cells. Nat Biotechnol 33: 543-548.

[8] Shin S, Kim SH, Park J-H, Lee JS, Lee GM (2022) Recombinase-mediated cassette exchange-based screening of a CRISPR/Cas9 library for enhanced recombinant protein production in human embryonic kidney cells: Improving resistance to hyperosmotic stress. Metab Eng 72: 247-258.

[9] Shin SW, Lee JS (2020) Optimized CRISPR/Cas9 strategy for homology‐directed multiple targeted integration of transgenes in CHO cells. Biotechnol Bioeng 117: 1895-1903.
